# Key RNA-binding domains in the La protein establish tRNA modification levels in *Trypanosoma brucei*

**DOI:** 10.1093/nar/gkaf594

**Published:** 2025-07-10

**Authors:** Lankani Gunaratne, Henry Moore, Nicholas Albaum, Ananth Casius, Jeremy Henderson, Alan Kessler, Eva Hegedűsová, Sneha Kulkarni, Henry Arthur, Robert L Ross, Zdeněk Paris, Richard Maraia, Todd M Lowe, Juan D Alfonzo

**Affiliations:** The Ohio State Biochemistry Program, The Ohio State University, Columbus, Ohio 43210, United States; The Center for RNA Biology, The Ohio State University, Columbus, Ohio 43210, United States; Department of Molecular Biology, Cell Biology and Biochemistry and the Brown RNA Center, 225 Dyer Street, Brown University, Providence, R I 02903, United States; Department of Biomolecular Engineering, University of California, Santa Cruz, CA 95064, United States; Department of Molecular Biology, Cell Biology and Biochemistry and the Brown RNA Center, 225 Dyer Street, Brown University, Providence, R I 02903, United States; The Center for RNA Biology, The Ohio State University, Columbus, Ohio 43210, United States; Department of Molecular Biology, Cell Biology and Biochemistry and the Brown RNA Center, 225 Dyer Street, Brown University, Providence, R I 02903, United States; New England Biolabs Inc, 240 County Road Ipswich, Massachusetts 01938, United States; Section on Molecular and Cell Biology, Eunice Kennedy Shriver National Institute of Child Health and Human Development (NICHD), National Institutes of Health (NIH), Bethesda, MD 20892, United States; Institute of Parasitology, Biology Centre, Czech Academy of Sciences, České Budějovice, 31 370 05, Czech Republic; Institute of Parasitology, Biology Centre, Czech Academy of Sciences, České Budějovice, 31 370 05, Czech Republic; The Center for RNA Biology, The Ohio State University, Columbus, Ohio 43210, United States; Thermo Fisher Scientific, Lexington, MA 04241, United States; Institute of Parasitology, Biology Centre, Czech Academy of Sciences, České Budějovice, 31 370 05, Czech Republic; Faculty of Science, University of South Bohemia, České Budějovice, 31 370 05, Czech Republic; Section on Molecular and Cell Biology, Eunice Kennedy Shriver National Institute of Child Health and Human Development (NICHD), National Institutes of Health (NIH), Bethesda, MD 20892, United States; Department of Biomolecular Engineering, University of California, Santa Cruz, CA 95064, United States; Department of Molecular Biology, Cell Biology and Biochemistry and the Brown RNA Center, 225 Dyer Street, Brown University, Providence, R I 02903, United States

## Abstract

The RNA-binding protein La is found in most eukaryotes, and despite being essential in many organisms, its function is not completely clear. *Trypanosoma brucei*, the causative agent of human African trypanosomiasis, encodes a ‘classical’ La protein (TbLa) composed of a La-motif, two RNA recognition motifs (RRM1 and RRM2α), a C-terminal short basic motif (SBM), and a nuclear localization signal (NLS). In *T. brucei*, like in most eukaryotes, position 34 of tRNA^Tyr^, ^-Asp^, ^-Asn^ and ^-His^ is modified with queuosine (Q_34_). The steady-state levels of queuosine-modified tRNA in the insect form (procyclic) of *T. brucei* can fluctuate dynamically depending on growth conditions, but the mechanism(s) controlling Q_34_ levels are not well understood. A well-established function of La is in precursor-tRNA 3′-end metabolism, but in this work, we demonstrate that La also controls Q_34_-tRNA levels. Individual domain deletions showed that while deletion of La motif or RRM1 causes dysregulation of Q_34_-tRNA levels, no other domain plays a similar role. We also show that La is important for the normal balance of several additional tRNA modifications. These findings are discussed in the context of substrate competition between La and modification enzymes, also highlighting subcellular localization as a key determinant of tRNA function.

## Graphical Abstract

**Graphical Abstract:**
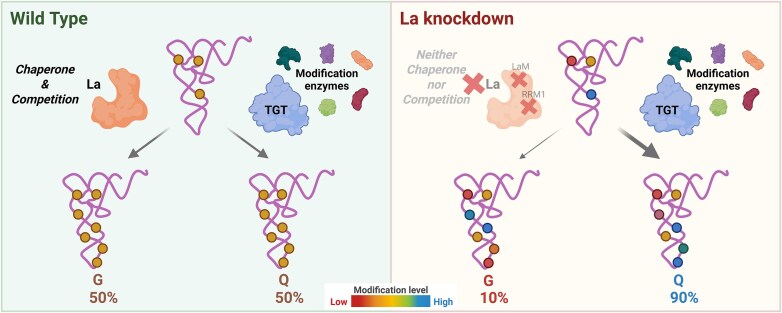

## Introduction

La is an RNA binding protein originally identified as an autoantigen in patients with chronic inflammatory disorders, systemic Lupus erythematosus and Sjögren's syndrome [1, 2]. La is found in most eukaryotes and is essential in most [3–6]. Among the many functions that have been ascribed to La, its role in pre-tRNA 3′-end metabolism is well established [7, 8]. One or more La-related proteins (LARPs) are also found in eukaryotic genomes, defined by the conserved RNA-binding domain named La motif (LaM) and an adjacent LARP family-specific RNA-recognition motif (RRM), which together form the so-called La-module characteristic of LARPs [3, 4, 9]. The number of LARPs vary among eukaryotic lineages [4], for example, humans encode seven; La (aka LARP3), as well as LARPs 1, 1B, 4/4A, 4B, 6, and 7, while some single-cell eukaryotes, including *T. brucei*, only encode “genuine” La (LARP3). LARPs expanded as a polyphyletic family during eukaryotic evolution involving major domain variations beyond the La-module [3, 4, 9]. For example, the La modules of different LARPs are appended to various types of RNA-binding or other functional domains and motifs: DM15 in LARP1 [3, 10], PolyA-binding protein-associated motif-2 (PAM2) in LARP4A/B and LARP1/B [11–14], the La and S1 associated motif (LSA) in LARP6 [3], the conserved region-2 (CR2) in LARP4A/B [3, 15], and the conserved basic region (CBR) found in LARP7 preceding its RRM2 [16]. Indeed, the various domains serve to diversify La/LARP function, which ranges from binding mRNA, to binding rRNA, and/or small non-coding RNAs including tRNAs [9, 16]. Finally, LARPs contain subcellular localization elements, which in the case of human La includes a nuclear retention element (NRE), nuclear export sequence (NES), nucleolar localization (NoLS), and nuclear localization sequence (NLS) [16–22].

The well-characterized high-affinity binding target of La is U_(1–3)_U-3′OH [23–25], formed by transcription of the T_(4–7)_termination signal specific to RNA polymerase III (Pol III), itself a hallmark of eukaryotes. La proteins bind newly synthesized Pol III transcripts, the most numerous and diverse of which are the pre-tRNAs [26–28]. By binding 3′-terminal U_(1–3)_U-3′OH, La shields Pol III transcripts from 3′-exonucleolytic digestion and untimely degradation or processing [29–31]. La's relationship with tRNA modifications is also notable, as some modifications contribute to tRNA folding. Human La-associated precursor-tRNAs show stoichiometric levels of modifications like m^1^A_58_ [2, 32, 33], which is involved in tRNA structure and function [34, 35]. Yeast La (Lhp1) binding to tRNA reduces the levels of m^2,2^G_26_ modification by Trm1 and prevents misfolding in tRNAs [36–38]. However, no direct physical interaction between the two protein has been observed, yet both can redundantly support structurally challenged tRNAs, highlighting their conserved roles as tRNA chaperones [36–42].

Beyond pre-tRNAs, La can associate with mature tRNAs, rRNAs and mRNAs species that bind by unknown mechanisms [43–46]. For example, human La binds fully end-matured tRNAs with high affinity via protein regions not involved in U_(1–3)_U-3′OH recognition [47]. Although the significance of La binding mature tRNAs is not fully clear, the reported association of the La protein with various tRNA species [45, 47] raises the question of whether La may also play a role in affecting tRNA modification levels.

In a previous report, we showed that in *Trypanosoma brucei* queuosine modification at the first position of the anticodon (Q_34_) in tRNA^Tyr^, ^-Asp^, ^-His^ and ^-Asn^ changes dynamically in response to alterations in the levels of certain amino acids in the media, consequent to increased dwell time of tRNA in the nucleus [48]. Since *T. brucei* tRNA guanine transglycosylase (TbTGT), the enzyme responsible for Q_34_ formation in tRNA is nuclear, we concluded that there is a competition between the rate of tRNA nuclear export and the ability of TbTGT to target tRNAs for modification [49, 50]. Indeed, downregulation of the tRNA exporter (TbMex67) increased tRNA dwell time in the nucleus, with concomitant increase in Q_34_ [51], to phenocopy the effect seen when cells are grown in the presence of specific, low amino acid levels [48].

Since *T. brucei* La (TbLa) and TbTGT are nuclear enzymes [49, 50, 52] and given the previous observations described above, we hypothesized that there may be competition for the tRNA substrates between TbLa and other tRNA-interacting factors, including modification enzymes. In this study, we show that downregulation of TbLa expression by RNA interference leads to an increase in the Q_34_ content of all four tRNAs that are targets of TbTGT without significant changes in transcript levels. By using a combination of genetic and biochemical approaches we dissected the contribution of each La domain to tRNA Q_34_ content, leading to the conclusion that the La module (LaM and RRM1) and, implicitly, tRNA binding are essential for regulating the levels of Q_34_. We also provide OTTR-seq and LC-MS/MS data showing that the effect of TbLa extends to additional tRNA modifications with varied effects; some modifications increase in the absence of TbLa, others decrease. This indicates that TbLa influences global tRNA modification levels by different mechanisms, but in the specific case of queuosine it helps maintain ∼50% steady state levels presumably needed based on translational decoding arguments [48, 50]. Conceptually, substrate competition may offer a rapid response to modification changes that goes beyond transcription and can easily be adapted to changes in environmental cues. Given the near-universal occurrence of La in eukaryotes, our findings likely extend beyond the *T. brucei* system.

## Materials and methods

### *T. brucei* cell culturing and generation of RNAi cell lines expressing TbLa mutants

Procyclic form of *Trypanosoma brucei* 29–13 cells were grown in SDM-79 media supplemented with 10% FBS and Hemin at 27°C. TbLa was knocked down using a p2T7-177 vector through expression of a double stranded RNA (dsRNA) targeting TbLa mRNA (Tb927.10.2370) via head-to-head oriented T7 promoters [53]. The dsRNA expression was induced by treating cells with 1 μg/ml of tetracycline [54]. The growth of the cultures was assessed through counting cells using a Neubauer hemocytometer. The region that was targeted by the La RNAi induction in the TbLa gene was recoded with synonymous codons to generate the TbLaRec gene (supplementary Fig. S3). pABPuro vector with Puromycin selection was used to express myc-TbLaRec and the myc-TbLaRec domain mutants in 29–13 cells expressing La RNAi. The Thermo Scientific Phusion site directed mutagenesis kit (Catalog: F541) was used to generate the TbLaRec domain mutants. All the vectors were transfected into *T. brucei* 29–13 cells via electroporation followed by selection using appropriate drugs [55]. The selected clones were analyzed via polymerase chain reaction (PCR) with genomic DNA, western blotting, and Reverse Transcription (RT)-PCR. Following oligos were used to generate the myc-TbLaRec mutants. LaMΔ: FP- GGAGGAAAGTGGGAGGTTCAAGTC, RP- TCCATCCAAACCGATCACCAAACC, RRM1Δ: FP- AGCTCAAAGACGAAAAATGGAGGAGC, RP- GTGATCGGTTTGGATGGATTCCGG, RRM2αΔ: FP- TCAGCTAACAACAGGAGCGGAAGG, RP- TGGCATAGGCGGAGTCTTTTTTTCAG, SBMΔ: FP- GGGCATAAACGCAGCAGAGAGTAAG, RP- GTTGTTAGCTGATTGCATAGCCCTGT).

### Reverse transcription—PCR

*T. brucei* cultures at log phase (2–8 × 10^6^ cells/ml) were used to extract total RNA using guanidinium thiocyanate–phenol–chloroform protocol as previously described [56]. RNA samples were treated with DNase I to remove genomic DNA. Around 2 μg of each RNA sample was subjected to reverse transcription and PCR using oligos specific to endogenous TbLa mRNA (FP1: CCGCTTGGAAGTAGCTATCGTGTAAGC, RP1: TTCACGTGACCGCTTGTGTCCT) and Recoded TbLa mRNA (For myc-TbLa Rec, myc-TbLa LaMΔRec and myc-TbLa RRM1ΔRec; FP2: GTTAAAAACCTCTGGCCAGTTGAAGAACA, RP2: GCTCCTGTTGTTAGCTGATTGCATAGC, For myc-TbLa RRM2αΔRec and myc-TbLa SBMΔRec; FP3: CCTGAAGGGGAAGATGGCAGAAAATG, RP3: TGGCATAGGCGGAGTCTTTTTTTCAG).

### APB gel electrophoresis and Northern blot analysis

Total RNA of *T. brucei* was isolated as previously described [56]. RNA was deacetylated in 0.1 M Tris-HCl at pH 9.0 for 30 min at 37°C prior to gel electrophoresis. Samples of 5 μg of RNA were denatured at 70°C and separated on 8% polyacrylamide gels containing 8M Urea, 0.5 % 3-aminophenyl boronic acid (APB) and 1x TAE [57]. Electrophoresis was carried out at 75 V for 5.5 h at 4°C. The RNA in gels were visualized using ethidium bromide staining for 10 min and then, electroblotted into Zeta probe nylon membranes for 2 h at 200 mA at 4°C according to the manufacturer's protocol (Bio-Rad). RNA was crosslinked to the membranes by UV irradiation for 1 min before hybridization. Northern blot hybridization was done according to Bio-Rad manufacturer's protocol. The blots that were hybridized with radiolabeled probes were exposed to a phosphorimager screen overnight and imaged using a Typhoon FLA 9000 scanner. The band intensities were calculated using ImageLab software (Bio-Rad). The blots were stripped and re-probed according to the manufacturers’ protocol. (Bio-Rad) Following oligonucleotides end-labeled with ^32^P were used to probe for the tRNA species of interest. (tRNA^Tyr^: CCTTCCGGCCGGAATCGAACCAGCGAC, tRNA^Asp^: CGGGTCACCCGCGTGACAGG, tRNA^His^: GGGAAGACCGGGAATCGAAC, tRNA^Asn^: GATTCGAACCAACGACCTGTAGGT, tRNA^Glu^: TTCCGGTACCGGGAATCGAAC).

### Fluorescence *in situ* hybridization (FISH)

*T. brucei* 29–13 cells were harvested on days 4, 5, and 6 post-La RNAi induction and washed twice with 1x Phosphate saline buffer (PBS). 600 000 cells resuspended in 4% paraformaldehyde/PBS solution were smeared onto poly-L-lysine coated microscope slides and fixed overnight. Slides were washed with 1x PBS and then permeabilized by dehydration in a series of solutions with increasing ethanol concentrations (50, 80, and 100%) for 3 min each. Next, the slides were prehybridized for 2 h in hybridization buffer (2% BSA, 5 × Denhardt's solution, 4 × SSC, 5% dextran sulfate, 35% deionized formamide, 10 U/ml RNase inhibitor). The hybridization was carried out in the hybridization buffer containing 10 ng/μl Cy3-labeled oligonucleotide probe (TbtRNA^Tyr-mature^: Cy3-AACCAGCGACCCTGTGATCTAC, TbtRNA^Asp^: Cy3-CGGGTCACCCGCGTGACAGG), in a humid chamber overnight at room temperature. Then the slides were washed with 4 × SSC with 35% deionized formamide, 2x SSC, and 1x SSC (for 10 min in each). The mounting medium supplemented with 4′,6-diamino-2-phenylindole dihydrochloride (DAPI) was added to the slides at the end. A confocal microscope Olympus FluoView™ FV1000 was used for imaging and Fluoview and ImageJ (NIH) software were used for processing the images.

### Immunofluorescence microscopy

*T. brucei* cultures were supplemented with 200 mM Mitotracker Red dye for 30 mins at 27°C to stain mitochondria in cells. Cells were harvested by spinning at 2300g for 5 min, washed twice with 1x PBS and resuspended in 1x PBS solution containing the fixative agent, 4% paraformaldehyde. Around 6 × 10^5^ cells were smeared onto a glass slide, air-dried, and washed with 1x PBS. Cells were permeabilized with methanol for 10 min at −20°C and rinsed with 1x PBS three times. Slides were blocked for 1 h using 5.5% fetal bovine serum (FBS) in 1x PBST (1x PBS with 0.1% Tween-20) followed by two washes with 1x PBS. Cells were incubated with anti-Myc Mouse primary antibody at 1:300 dilution in 3% FBS in 1x PBST for 1 h followed by washing thrice with 1x PBST and twice with 1x PBS. Cells were incubated with Alexa-488 conjugated to goat derived anti-Mouse secondary antibody at 1:1000 dilution in 3% FBS in 1x PBST for 1 h followed by washing thrice with 1x PBST and twice with 1x PBS. Cellular DNA was stained with 1x PBS containing 300 nM DAPI for 2 min followed by two washes with 1x PBS followed by air-dry. The smear was mounted with Vectashield, covered with a coverslip and the edges were sealed with nail polish. The cells were imaged using a Nikon Ti microscope and images were processed using ImageJ (NIH) and Hyugens Essentials 3.4 software.

### Recombinant his-TbLa purification

The coding sequence of TbLa was cloned into the expression vector pET15b and the TbLa domain mutants (LaMΔ and RRM1Δ) in pET15b were constructed as described above. Recombinant His(8x)-TbLa protein and mutants were expressed in *Escherichia coli* BL21 cells. For protein purification, 1.5 L cultures were grown at 37°C while shaking at 200 rpm, until OD_600_ reached 0.6–0.8. Then protein expression was induced by adding isopropyl β-d-1-thiogalactopyranoside (IPTG) to a final concentration of 0.5 mM and cultures were grown at room temperature (∼25°C) overnight while shaking. Harvested cell pellets were resuspended in lysis buffer (20 mM Tris pH 8.0, 1 M NaCl, 10% glycerol, 20 mM Imidazole, 1x protease inhibitor cocktail, 1x PMSF and 0.01% NP40) and lysed by sonication with a Sonifier 450. Lysate was centrifuged at 40 000 x g for 30 min, followed by 50 000 x g for 30 min to remove cellular debris. Resultant supernatant was injected through a 5 mL HisTrap HP column (Cytiva, Ni^2+^-nitrilotriacetic acid (NTA) column) via AktaPure FPLC. Bound material was washed with lysis buffer and a wash buffer (20 mM Tris pH 8.0, 100 mM NaCl, 10% glycerol, 50 mM Imidazole, 1x protease inhibitor cocktail, and 1x PMSF), 20 column volumes in each. Bound protein was eluted (20 mM Tris pH 8.0, 100 mM NaCl, 10% glycerol, 600 mM Imidazole, 1x protease inhibitor cocktail, and 1x PMSF). Peak fractions were pooled, and buffer was exchanged using the size exclusion column (HiLoad^TM^ 16/600 Superdex^TM^ 75 pg) to a storage buffer (50 mM Tris pH 8.0, 50 mM NaCl, 14% glycerol, 1x protease inhibitor cocktail, and 1x PMSF). Final isolated protein was aliquoted and stored at −80°C.

### Electrophoretic mobility shift assay (EMSA)

Native tRNA^Tyr^ lacking the Q modification was extracted from total RNA preps of TbTGT2Δ cells using biotinylated oligos following the protocol published by Spears *et al.* in 2011 [58]. The tRNA was end-labeled with γ-(^32^P) ATP using T4 polynucleotide kinase (PNK) (NEB: M0201L) and subsequent radiolabeled mature tRNA^Tyr^ was gel-purified. The radiolabeled tRNA was denatured at 70°C for 3 min, then folded by slowly cooling down at room temperature, and incubated at 37°C for 15 min. Approximately 4000 CPM of folded tRNA (∼0.6 nM) was incubated on ice for 30 min with His-TbLa solutions of varying concentrations (0–1.6 μM) in 1x HKM buffer (50 mM HEPES pH 7.5, 1 mM MgCl_2_, 5 mM KCl) in a total volume of 10 μl. Next, 5 μl of 50% glycerol was mixed with each solution and the products were separated on a 6% nondenaturing polyacrylamide gel at 90 V for 2.5 h. Gels were vacuum dried and exposed to a phosphorimager screen overnight. A Typhoon FLA 9000 phosphorimager was used to visualize the screen and band intensities were measured using ImageLab software. The fraction of bound tRNA was calculated using the equation tRNA_complex_/ (tRNA_free_ + tRNA_complex_) and plotted against the concentration of TbLa in each reaction. The data points were fit to a Hill plot and the apparent dissociation constants (K_Dapp_) were estimated by nonlinear regression using Graphpad Prism software.

### Total nucleoside analysis by LC-MS/MS

#### Sample preparation

Total RNA extracted from La RNAi induced and uninduced *T. brucei* cells were separated on 8% urea-polyacrylamide gels and visualized by ethidium bromide staining under ultraviolet light. The band at the size of tRNA (∼75 bp) was excised from the gel and RNA was eluted by incubating it in a solution of 0.3 M Sodium Acetate (pH 5.2) overnight. The eluted tRNA was concentrated by ethanol precipitation and was digested enzymatically to monomers as previously reported [59]. Briefly, the purified tRNA was denatured by heating at 100°C then quickly chilled in an ice water bath. 1/10 volume of 0.1 M ammonium acetate, 1U (units) of nuclease P1 (Sigma Aldrich, St. Louis MO.) was added to the tRNA and incubated at 45°C. After 2 h, 1/10 volume of 1 M ammonium carbonate was added with 1.2 × 10^–4^ U of snake venom phosphodiesterase (Worthington Biochemicals, Lakewood, NJ.) and 0.003 U FastAP (Thermo Fisher Scientific, San Jose, CA.), and incubated at 37°C for 2 h. The digested nucleosides were then dried in speed vac and resuspended in 10 μl of mobile phase A for analysis.

#### Liquid chromatography (LC) method

Around 1 μl of each sample was injected onto a microbore C18 UHPLC column, 0.5 × 100 mm, 1.7 μm (Mac-Mod Analytical, Chadds Ford, PA.) with the Vanquish Neo UHPLC system (Thermo Fisher Scientific, San Jose, CA.) at a flow rate of 30 μl min^-1^ using an ammonium acetate/acetonitrile buffer system. Mobile Phase A was 5 mM Ammonium acetate pH 5, Mobile Phase B was 60% MPA and 40% ACN. Gradient starts at 0% B held for 0.1 min then increasing to 1.5% at 7.2 min, 3% at 9.7 min, 5% at 15 min, 25% at 18 min, 50% at 20 min, 75% at 23 min, and held for 1.5 min before increasing to 90% at 30 min, 99% at 35 min then returning to 0% for 10 column volume re-equilibration.

#### Mass spectrometry (MS) method

Acquisition was performed on an Orbitrap Ascend BioPharma Tribrid mass spectrometer (Thermo Fisher Scientific, San Jose, CA.) running Xcalibur 4.6. For all experiments, data were collected in small molecule application mode. Full scan at a 60 000 orbitrap resolution with a scan range of 240−600 *m/z* was used. AGC target was set to standard, microscans was 1, the RF lens was 35% and maximum injection time was auto. MS2 was performed with decision tree method involving CID (collision induced dissociation) and HCD (higher energy collisional dissociation) in orbitrap with a Resolution of 30000. For CID collision energy of 40%, activation time of 10 ms and activation q of 0.25 was used. For HCD, 60 V of energy, activation time of 100 ms and microscans of 1 was used.

#### Data analysis

Data were processed using Compound Discoverer 3.1 (Thermo Fisher Scientific, San Jose, CA.) as previously described [60]. The area under the peaks for each modification was added up and internally normalized to the Cytosine level in each sample. The value of each modification in different samples was divided by the average values of RNAi uninduced control to calculate the fold difference between RNAi induced and uninduced samples. Unpaired two-tailed t-test with Welch correction was used to determine the statistical significance.

### Ordered two-template relay tRNA sequencing (OTTR-seq)

Total RNA extracted from La RNAi-induced and uninduced *T. brucei* cultures was incubated with T4 PNK (NEB, M0201L) to resolve 2′-3′ cyclic phosphate ends as described previously [61], and then deacylated by bringing the reaction to pH 9.0 for 30 min at 45°C using 100 mM Na_2_B_4_O_7_ [62]. Treated RNA was recovered using the RNA clean and concentrate kit (Zymo research, Irvine, CA, USA, R1013). OTTR-seq libraries were prepared according to previously described methods [63] with modifications. Briefly, 40 ng of total RNA was used as input. RNA was labeled for 2 h at 30°C using only dideoxy ATP (ddATP). Similarly, cDNA synthesis was conducted using only the RNA-DNA primer duplex with + 1T overhang. cDNA was recovered using the Minelute reaction cleanup kit (Qiagen, 28 204), then size selected on a 1 × Tris-borate-EDTA (TBE, 89 mM Tris, 89 mM boric acid, 2 mM EDTA, pH 8.3) buffer, 8M urea, and 9% acrylamide gel to remove adapter dimers. cDNA was recovered by diffusion at 70°C for 1 h with shaking in a solution of 10 mM Tris-HCl pH 8, 300 mM NaCl, 1 mM EDTA, and 0.25% SDS, followed by precipitation in 66% ethanol with 0.6 M ammonium acetate and 1 μg/mL glycoblue (Thermo Fisher, AM9515). cDNA was amplified with Q5 polymerase (NEB, M0493L) for 13 cycles using the Illumina NEBNext sRNA index adapters (NEB, E7600S) then size selected on a 1 × TBE, 6% acrylamide gel to remove remaining adapter dimer. Libraries were recovered from the gel by diffusion at 70°C for 1 h with shaking in a solution of 10 mM Tris-HCl pH 8, 300 mM NaCl, and 1 mM EDTA, followed by precipitation in 70% ethanol with 80 mM sodium acetate and 1 μg/mL glycoblue (Thermo Fisher, AM9515). The recovered libraries were quantified using the qubit dsDNA High Sensitivity kit (Invitrogen, Q32854), Agilent High Sensitivity DNA bioanalyzer kit (Agilent, 5067–4626), and finally by qPCR. qPCR was conducted using PrimeTime Gene Expression Master Mix (IDT, 1 055 772) according to a protocol published online (https://www.protocols.io/view/illumina-truseq-library-quantification-with-qpcr-p-6qpvrddpzgmk/v1). Libraries were sequenced on the NextSeq 1000/2000 platform to generate 100 bp single end reads. Each sample had a minimum of four million reads per sample.

Raw FASTA files were trimmed using Cutadapt with options -j 8 -m 15 -a GATCGGAAGAGCACACGTC. Reads were further processed to remove an 8nt UMI using umi_tools extract with options –extract-method = string –bc-pattern = NNNNNNN. Trimmed reads were processed through the tRNA Analysis of eXpression pipeline (tRAX) [64], using a custom reference database generated for the *T. brucei* Lister 427 genome assembly. Resulting BAM files were split into reads above or below 60 bp in length, and those above 60 bp were processed through the tRAX pipeline again (using the –lazyremap option) to analyze the expression of mature tRNA transcripts. Modifications in mature tRNA were inferred from the presence of misincorporations at the site of known modifications in other eukaryotes (PMID: 38 015 436), using the -coverage.txt file output by tRAX and applying a conservative misincorporation cutoff of 10%. Changes in modification abundance were estimated by log2-fold change in misincorporation rate. Statistical significance of changes in misincorporation rate were calculated via a two-tailed T-test and adjusted for multiple testing via Benjamini/Hochberg procedure with a family-wise error rate of 0.001.

## Results

### Downregulation of TbLa expression leads to increased Q-tRNA levels

**Figure 1.**
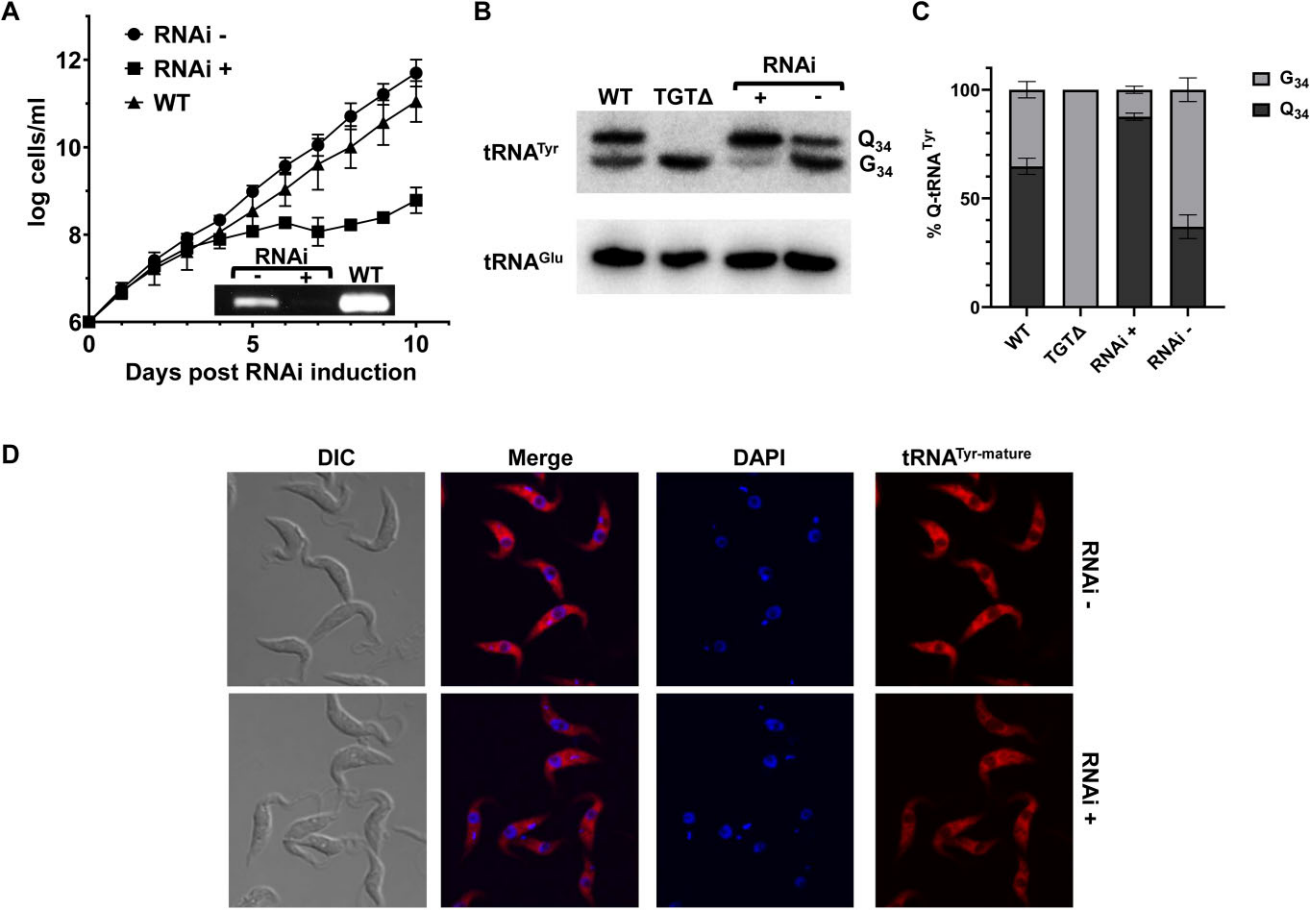
Knockdown of TbLa increases Q_34_ levels in tRNA. (**A**) Growth curves of *T. brucei* wild type (WT, triangles), RNAi uninduced (RNAi-, circles) and TbLa RNAi induced cells (RNAi+, squares). The inset shows RT-PCR using total RNA extracted on day 5 of the growth curve. RNAi was induced by addition of tetracycline to the media (as described in the “Materials and methods” section). (**B**) An APB-northern blot with radioactive oligonucleotide probes specific for tRNA^Tyr^ and tRNA^Glu^ and total RNA samples as above from WT, TGT knockout (TGTΔ), TbLa RNAi- and RNAi + cells, tRNA^Glu^ serves as an RNA input normalization control_._ (**C**) Graphical representation of percent of Q modified tRNA^Tyr^ shown in from (**B**). (**D**) Fluorescence *in situ* hybridization of TbLa RNAi uninduced (RNAi-) and induced (RNAi+) cells, probed for mature tRNA^Tyr^ with a Cy3-labeled oligonucleotide probe specific for the spliced anticodon arm. DIC refers to the phase contrast image, DAPI stains nuclear and kinetoplastid (mitochondrial) DNA and merge shows the overlapped images of DAPI and Cy3 probes.

Previous studies showed that just like in humans, the La protein is essential in *T. brucei* [6, 30]. Most recently, a connection between La and the modification enzyme Trm1 in *S. pombe* was reported, demonstrating a non-catalytic function for Trm1 as an RNA chaperone, with synergistic regulation of Trm1 methyltransferase activity via La binding to pre-tRNA populations [38]. In this report we explore the connection between *T. brucei* (TbLa) and tRNA modifications at a global scale, given the nuclear colocalization of TbLa with various tRNA modification enzymes, and the ability of TbLa to bind tRNAs [36, 47]. For this, we downregulated TbLa expression in *T. brucei* by establishing clonal lines in which a portion of the TbLa coding sequence was cloned into an RNAi plasmid vector under the control of a tetracycline operator. In this system RNAi is tetracycline inducible [54]. Upon tetracycline addition to the growth media, we observed a significant slowdown in cell growth as compared to a wild-type cell line or an uninduced control (where tetracycline was omitted from the growth media) (Fig. 1A). Downregulation of TbLa expression was further confirmed by reverse transcription followed by polymerase chain reaction (RT-PCR) with oligonucleotides specific for the TbLa mRNA, which showed a significant reduction in the steady-state TbLa mRNA levels as compared to RT-PCR with RNA samples from wild-type and/or uninduced cells (Fig. 1A). These results recapitulate previous observations about the importance of La for *T. brucei* growth [6, 30].

Prior to this work, we showed that tRNA-guanine transglycosylase (TGT), the enzyme responsible for Q_34_ formation in tRNA, is uniquely nuclear in *T. brucei* [49, 50]. Thus, we used TGT and the Q system to explore what effect, if any, downregulation of TbLa has on Q_34_ levels. For this, total RNA was extracted from RNAi-induced, wild-type and uninduced cells and Q_34_ content in tRNA was assessed by separation on denaturing acrylamide gels containing 3-aminophenylboronic acid (APB) [57]. Because APB has affinity for *cis-*diols, such as those present in Q_34_-containing tRNAs, it leads to an electrophoretic mobility shift if Q_34_ is present [65], which we detect by northern blots using oligonucleotide probes specific for different tRNA species. These experiments showed that under “normal” growth conditions the steady-state levels of Q_34_ in tRNA^Tyr^ range from 40 to 60% as shown previously (Fig. 1B and C) [48, 49]. To show that the observed band shift is indeed due to the presence of Q_34_, we also probed total RNA isolated from a knockout of TbTGT2, the catalytic subunit of the heterodimeric TbTGT enzyme (TGTΔ). In this case no Q_34_ modified tRNAs were detected and this sample serves as a reference control in this and subsequent experiments. When expression of TbLa was downregulated by RNAi, the levels of Q_34_ in tRNA increased to nearly 90%, while the Q_34_ levels in RNA from uninduced cells remained within the 40–60% range (Fig. 1B and C). Similar increases upon TbLa downregulation were also observed with the other Q_34_-containing tRNAs (tRNA^Asp^, tRNA^His^, and tRNA^Asn^, supplementary Fig. S1). These results indicate that TbLa plays an important role in regulating Q_34_ levels of tRNA in *T. brucei*.

### High Q_34_ levels are not due to increased tRNA dwell time in the nucleus

We next explored possible mechanisms that lead to the observed increase of Q_34_ in tRNA in the absence of TbLa. In a previous report, we showed increased Q_34_ levels in tRNA upon reducing the levels of certain amino acids in the media including tyrosine [48], or upon downregulation of the tRNA exporter TbMex67 [51]. We established in both cases that the observed higher levels of Q_34_-content in tRNA were partly due to increased dwell time of particular tRNAs in the nucleus [48, 51], indicating that there is competition between the rate of nuclear export and the rate of Q_34_ formation in the nucleus. Therefore, we hypothesized that TbLa is required for efficient tRNA nuclear export, where absence of TbLa increases the nuclear dwell time of tRNA and consequently increases Q_34_. To test this hypothesis, we analyzed the localization of mature tRNA^Tyr^ in *T. brucei* cells by fluorescent *in situ* hybridization (FISH) using a fluorescently labeled oligonucleotide probe specific for the spliced anticodon arm of tRNA^Tyr^. We compared the localization of mature tRNA^Tyr^ between cells where TbLa expression is normal or as downregulated by RNAi. These experiments revealed no difference in localization and no evidence of increased dwell time in the nucleus in the absence of TbLa, formally refuting the hypothesis that the observed increase in Q_34_ levels is due to some role of TbLa in tRNA nuclear export (Fig. 1D). Q_34_-tRNA species that are not encoded with an intron, for example tRNA^Asp^, also localized similarly to tRNA^Tyr^ (Supplementary Fig. S2), with no evidence of increased nuclear dwell time in the absence of TbLa. Therefore, the observed increase in Q_34_ must occur by a different mechanism.

### The La motif and RRM1 domains are important for maintaining normal Q_34_ levels

**Figure 2.**
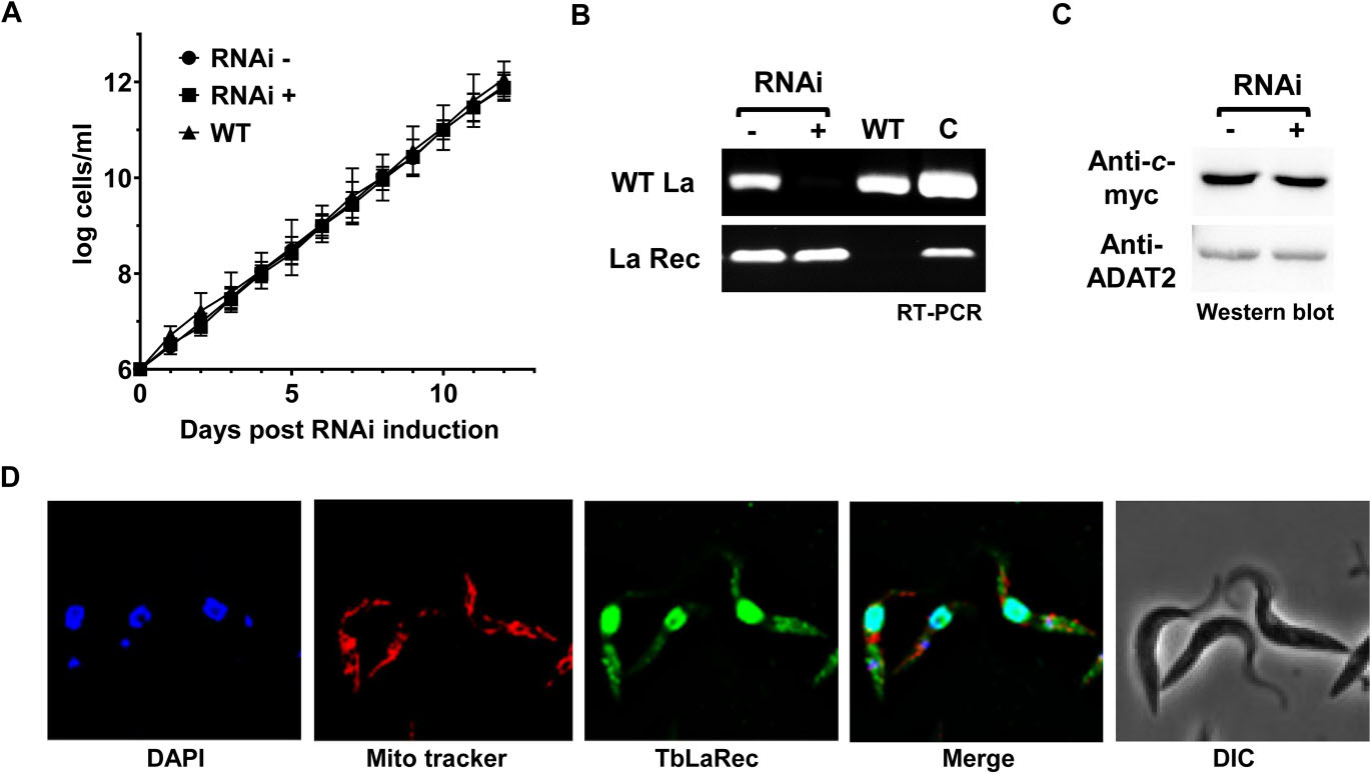
Recoded La rescues the growth defect caused by the knockdown of endogenous TbLa protein. (**A**) Growth curves of wild type (WT), uninduced (RNAi-) and induced (RNAi+) of endogenous TbLa cells expressing a constitutive myc-tagged recoded version of TbLa (myc-TbLaRec). (**B**) Agarose gels showing RT-PCR of endogenous TbLa mRNA (WT La) and recoded myc-TbLa mRNA (La Rec) using total RNA extracted on day 5 of the growth curve (as in Fig. 1). The “WT” lane indicates the RT-PCR of *T. brucei* total RNA from WT (29–13) cells and the “C” lane indicates the PCR using genomic DNA as the positive control. In this experiment RNAi refers to downregulation of endogenous TbLa. (**C**) Western blot with anti-*c*-myc antibodies and total protein extracted from uninduced (RNAi-) and induced (RNAi+) cells expressing myc-TbLaRec. The blot was stripped and probed for ADAT2 using anti-ADAT2 polyclonal antibodies as the loading control. (**D**) Immunofluorescence microscopy of TbLa RNAi cells expressing myc-TbLaRec, where Alexa 488-conjugated antibodies were used to detect myc-TbLaRec, while Mitotracker stains the mitochondria and DAPI nuclear and kinetoplast (mitochondria) DNA, merge shows the overlapping stains and DIC refers to a phase contrast image.

**Figure 3.**
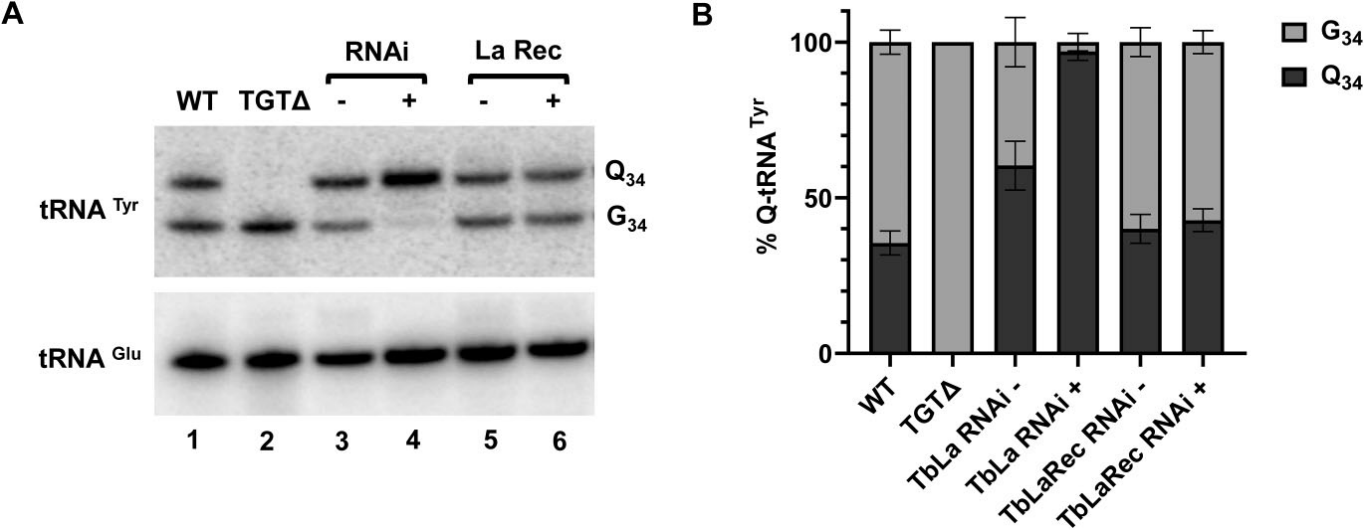
Recoded La protein restores Q_34_ levels in tRNA. (**A**) Levels of Q_34_ in tRNA^Tyr^ determined by APB-northern blot. ^32^P-oligonucleotide probes specific for tRNA^Tyr^ or tRNA^Glu^ were incubated with membranes from APB-PAGE separated total RNA isolated from WT, TbTGT knockout (TGTΔ), TbLa RNAi uninduced (−) and induced cells (+) (lanes 1–4), as well as the endogenous TbLa RNAi cell line constitutively expressing the recoded version of TbLa (LaRec) with RNAi uninduced (−) or induced (+) (lanes 5 and 6). (**B**) Graphical representation of the percentage of Q_34_ modified tRNA^Tyr^ as shown in (**A**). Error bars obtained by measurement from three independent biological samples.

An alternative hypothesis is that tRNA binding by TbLa in the nucleus competes with TbTGT, thus reducing the availability of tRNA substrates to be modified. In this scenario, the binding domains of TbLa should be important to maintain normal Q_34_ levels. To test this hypothesis, we first generated a cell line expressing a recoded version of TbLa (TbLaRec), in which TbLa codons were swapped with synonymous codons in the region that is targeted by RNAi (Supplementary Fig. S3). The resulting recoded TbLa (TbLaRec) mRNA is thus impervious to RNAi of endogenous TbLa, yet generates an otherwise wild-type protein with identical amino acid sequence to endogenous TbLa. The TbLaRec was expressed from a plasmid in the endogenous La RNAi cells allowing degradation of the endogenous TbLa mRNA by RNAi and simultaneous expression of TbLaRec. Indeed, this construct could rescue the growth defect generated by La RNAi, upon addition of tetracycline to the media (Fig. 2A). Degradation of the endogenous TbLa mRNA in this background was confirmed by RT-PCR (Fig. 2B), while the mRNA for TbLaRec remains stably expressed (Fig. 2B). Expression of the recoded protein was also detected by western blots with antibodies against a Myc-tag placed at the N-terminus of the recoded protein (Fig. 2C). La proteins, including TbLa, are mainly targeted to the nucleus with small amounts found in the cytoplasm [22, 52, 66]. To confirm the proper localization of TbLaRec, we used anti-Myc antibodies for immunofluorescence microscopy, revealing that TbLaRec is localized to the nucleus (Fig. 2D) similar to what has been reported for the native protein [52]. We also isolated RNA from the La RNAi cells expressing TbLaRec when RNAi is induced and uninduced. The resulting total RNA was then analyzed by APB-northern blot hybridization as before and the levels of Q_34_ in tRNA^Tyr^ were determined while comparing RNAi induced versus uninduced cells. Normal levels of Q_34_ are restored with TbLaRec when endogenous La is downregulated by RNAi (Fig. 3A), in contrast to the almost 100% Q_34_ increase in tRNA^Tyr^ samples from the parental La RNAi induced cells (Fig. 3A and B). These results show that TbLaRec completely rescues the endogenous TbLa function.

**Figure 4.**
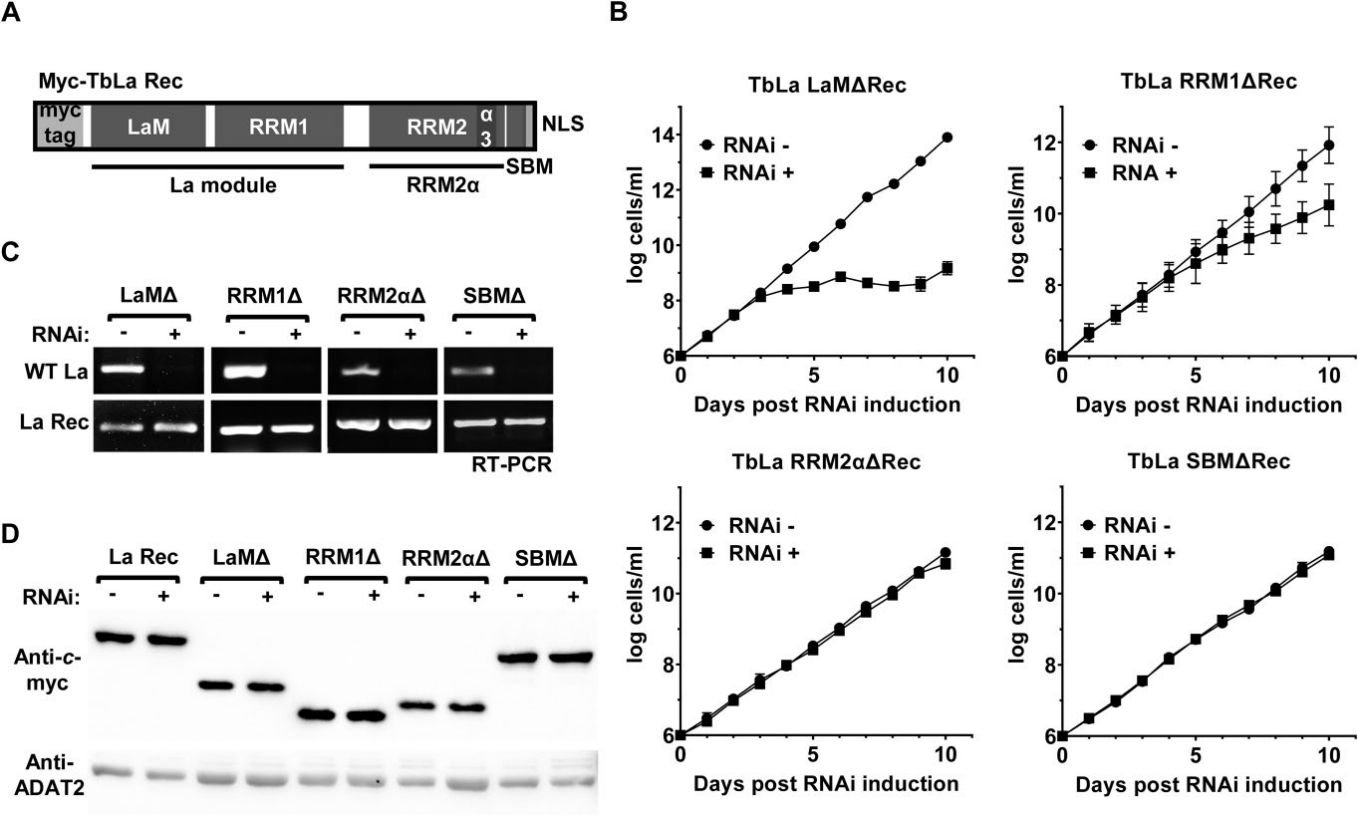
Only LaM and RRM1 domains of TbLa are important for normal cell growth. (**A**) Schematic representation of each domain of myc-TbLaRec, showing the La motif (LaM), RNA recognition motif 1 (RRM1), RNA recognition motif 2, which includes the α3 helix (RRM2α), Short basic motif (SBM), and the nuclear localization signal (NLS). (**B**) Growth curves of TbLa RNAi uninduced (RNAi -) and induced (RNAi +) cells constitutively expressing TbLaRec domain deletion mutants as indicated. (**C**) Agarose gels showing RT-PCR of endogenous TbLa mRNA (WT La) and recoded myc-TbLa mRNA (La Rec) using total RNA extracted on day 5 of the growth curves shown in (**B**). (**D**) Western blot of total protein extracted from RNAi uninduced (-) and induced (+) cells expressing each myc-TbLaRec mutant. The blot was stripped and probed for ADAT2 using anti-ADAT2 polyclonal antibodies as the loading control.

Using the TbLaRec cell line, we next explored the contribution of the different RNA binding domains of TbLa to regulation of Q_34_ levels. Previous work showed that the La module is responsible for both tRNA and UUU-3′OH binding [47, 67]. *In vitro*, the RRM2α domain binds structured RNAs [68] and the SBM is involved in 5′ triphosphate binding in pre-tRNA [8, 69]. Although the general function of these motifs is not well-established, it has been proposed that RRM2α could be functionally significant due to the correlation between the presence of this domain and the essentiality of La proteins across different species including humans [8]. The most conserved RNA binding domains of TbLa, the LaM (between residues 5–90) and RRM1 (between residues 96–192) have been structurally and biochemically characterized and are essential for pre-tRNA-UUU-3′OH binding although their ability to bind mature tRNA has not been tested before [67, 70]. While the RRM2α (between residues 213–314) and SBM (between residues 315–330) domains of TbLa have not been biochemically characterized, the high sequence conservation of these domains with other well-characterized La proteins, like human La, has allowed the bioinformatic prediction of location and secondary structure of these domains [9]. We generated a series of cell lines expressing various versions of TbLaRec with each of the aforementioned RNA binding domains deleted (Fig. 4A). These were expressed in the endogenous La RNAi cells. Upon addition of tetracycline to the media to induce RNAi of endogenous TbLa, we observed that neither the recoded LaMΔ mutant nor the RRM1Δ mutant could fully rescue the growth defect caused by TbLa downregulation while the RRM2αΔ and SBMΔ deletions showed complete growth rescue (Fig. 4B). Both the expression of the recoded mutants and degradation of the endogenous TbLa mRNA were confirmed by RT-PCR (Fig. 4C). To rule out the possibility that the lack of rescue was due to poor expression and/or stability of some of the mutants, we also performed western blots, which after normalization to a control protein showed no significant differences in the relative levels of any of the mutants as compared to that of the recoded control (Fig. 4D). Altogether, these results suggest that LaM and RRM1 domains are essential for TbLa function, while RRM2α and SBM domains are not, thus, ruling out the importance of the two C-terminal domains for TbLa essentiality.

**Figure 5.**
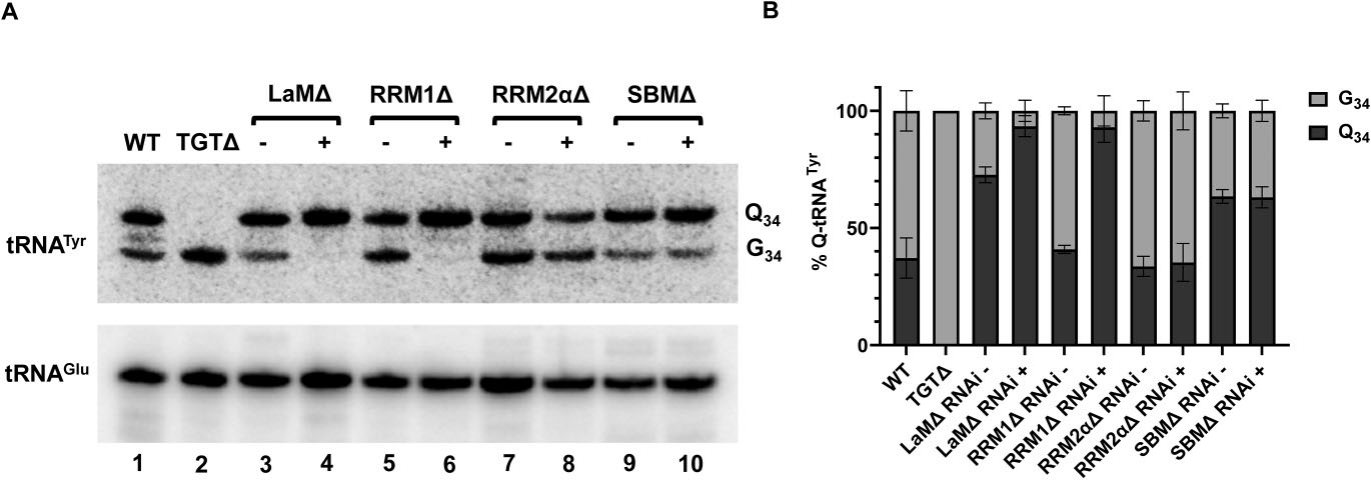
The key RNA-binding domains of TbLa (LaM and RRM1) are important for maintaining normal Q_34_ levels. (**A**) APB-northern blot with radioactive oligonucleotide probes specific for tRNA^Tyr^ and tRNA^Glu^ and total RNA from WT, TGT knockout (TGTΔ) (lanes 1 and 2), and the endogenous TbLa RNAi cell line constitutively expressing deletion mutants of each domain of TbLaRec as indicated: La motif (LaM) (lanes 3 and 4), RNA recognition motif 1 (RRM1) (lanes 5 and 6), RNA recognition motif 2, which includes the α3 helix (RRM2α) (lanes 7 and 8), Short basic motif (SBM) (lanes 9 and 10) when TbLa RNAi is uninduced (−) and RNAi induced (+). The signal from tRNA^Glu^ served as a normalization control. (**B**) Graphical representation of the percentage of Q_34_ modified tRNA^Tyr^ as shown in (**A**). Error bars obtained by measurement from three independent biological samples.

We then isolated total RNA from each recoded mutant to assess the contribution of each domain to the regulation of Q_34_ levels by APB-northern blot hybridization. It showed that neither the LaMΔ nor the RRM1Δ recoded mutants could restore normal Q_34_ levels, while Q_34_ remained at wild-type levels in the cells expressing the RRM2αΔ or the SBMΔ mutants (Fig. 5A and B).

**Figure 6.**
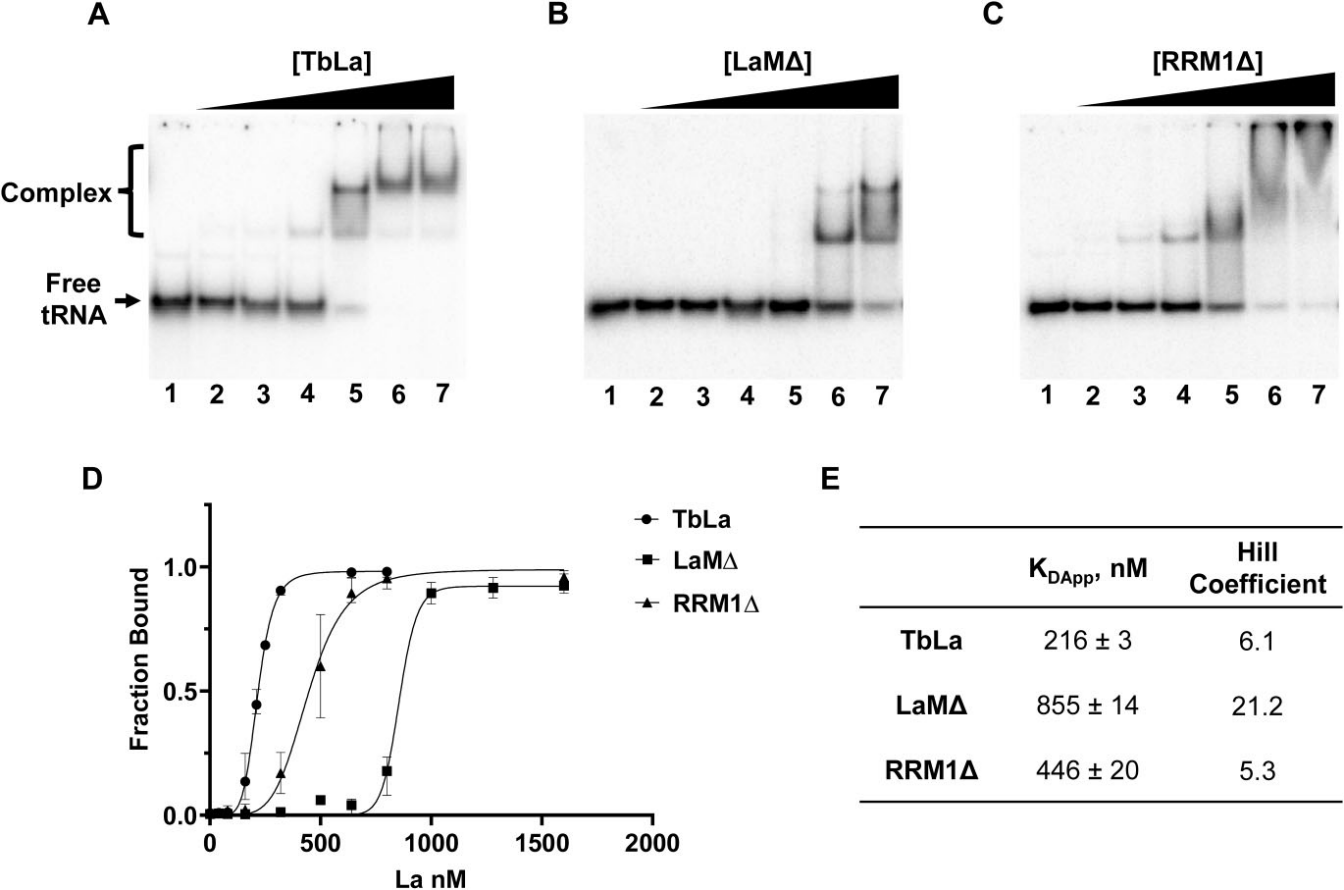
LaM and RRM1 are important for tRNA binding *in vitro*. Electrophoretic mobility shift assay (EMSA) performed on native, mature tRNA^Tyr^ isolated from *T. brucei*, which was T4 PNK 5′ ^32^P-end labeled prior to incubation with increasing concentrations of TbLa variants (**A**) wild type (**B**) LaM domain mutant (LaMΔ), or (**C**) RRM1 domain mutant (RRM1Δ). Protein-tRNA complexes were separated from unbound tRNA (free tRNA) on non-denaturing polyacrylamide gels. In all cases, lane 1 contains a no protein control, and lanes 2–7 contain TbLa, or domain deletion mutants at 40, 80, 160, 320, 640, and 800 nM, respectively. (**D**) Plotts of fraction bound tRNA graphed against increasing concentration of TbLa (circles), LaMΔ (squares), or RRM1Δ (triangles). (**E**) The apparent dissociation constants (K_Dapp_) expressed in nM, and hill coefficients for TbLa and mutants derived from graph (**D**). All the EMSAs, plots, and calculations are representative of three independent replicates.

To confirm that the deletions of LaM and RRM1 affect tRNA binding, as observed for other organisms [47], we recombinantly expressed those domain deletion mutants in *E. coli* and tested binding of each mutant to mature tRNA^Tyr^ by Electrophoretic Mobility Shift Assays (EMSAs) while comparing their binding behavior to reference wild-type TbLa protein. We found that while the apparent dissociation constant (K_Dapp_) for wild-type TbLa is 216 ± 3 nM, binding affinity was reduced with both mutants, with a K_Dapp_ of 855 ± 14 nM and 446 ± 20 nM for LaMΔ and RRM1Δ respectively (Fig. 6D and E). Interestingly, in all cases we observed a sigmoidal curve for binding. We thus transformed the data into a Hill plot, and in all cases positive cooperativity was observed (hill coefficients > 1), with highest cooperativity imparted by the LaM domain deletion. This observation suggests that these domains contribute to tRNA interaction despite not being previously reported as required for tRNA binding [67]. Although we do not currently fully understand the basis nor the importance of cooperativity, these results confirm that both LaM and RRM1 domains significantly contribute to mature tRNA binding by TbLa. Further, these results support our hypothesis that TbLa may regulate Q_34_ levels in tRNA through direct binding via LaM and RRM1 domains. It should be noted that at least in the case of tRNA^Tyr^, substrate recognition does not involve a tRNA UUU-3′OH motif, as tRNA^Tyr^ processing requires 3′ end-maturation, transit to the cytoplasm to be spliced, and finally return to the nucleus as an end-matured spliced molecule via retrograde transport to get Q_34_ [49, 51].

### TbLa globally impacts tRNA modifications

**Figure 7.**
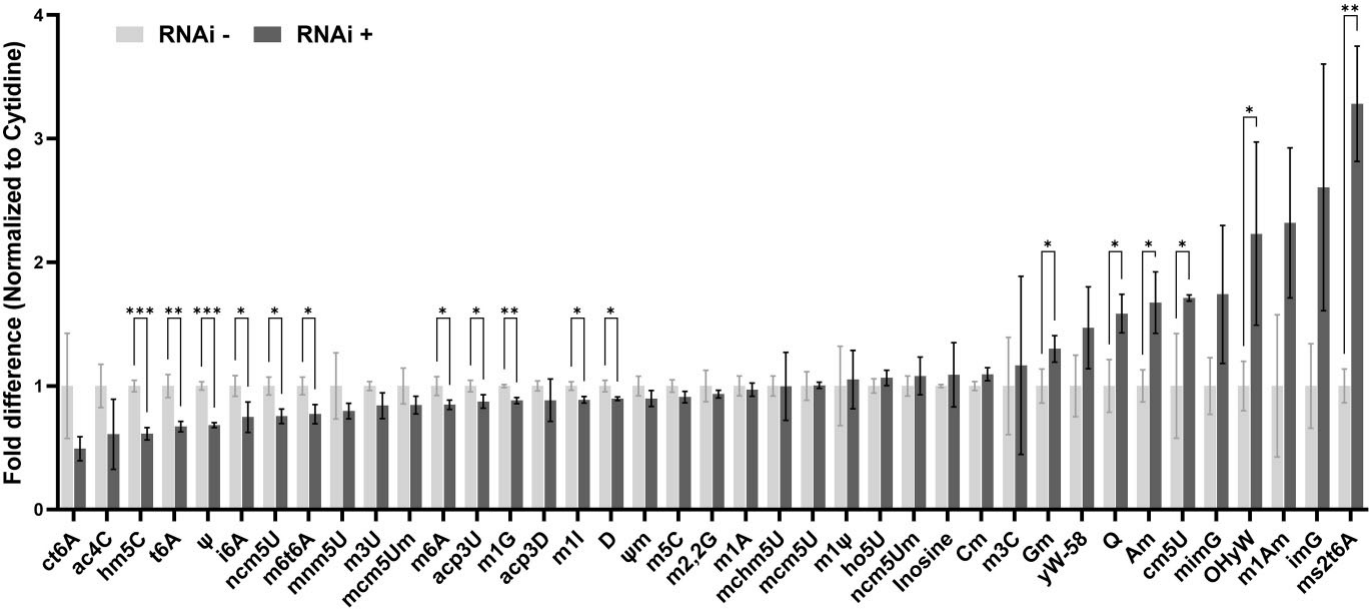
Global tRNA modification changes upon TbLa downregulation are not limited to Q_34_. Total tRNA samples from uninduced (RNAi-) and induced (RNAi+) were digested to nucleosides and analyzed by LC-MS/MS. Measurements were performed on triplicate biological samples. To calculate the fold difference, the raw values of each sample, were internally normalized to the values of Cytidine in each sample. Each sample quotient was then divided by the triplicate average of RNAi- samples. Asterisks (*) denote the *P*-value thresholds calculated using two-tailed unpaired t-test; *P < 0.05, **P < 0.01, ***P < 0.001 and ****P < 0.0001. (ct^6^A: Cyclic *N*^6^‐threonylcarbamoyladenosine, ac^4^C: *N*^4^‐acetylcytidine, hm^5^C: 5‐Hydroxymethylcytidine, t^6^A: *N*^6^‐threonylcarbamoyladenosine, ψ: pseudouridine, i^6^A: *N*^6^‐isopentenyladenosine, ncm^5^U: 5‐Carbamoylmethyluridine, m^6^t^6^A: *N*^6^‐methyl‐*N*^6^‐threonylcarbamoyladenosine, mnm^5^U: 5‐Methylaminomethyluridine, m^3^U: 3‐Methyluridine, mcm^5^Um: 5‐Methoxycarbonylmethyl‐2′‐O‐methyluridine, m^6^A: *N*^6^‐methyladenosine or 6‐methyladenosine, acp^3^U: 3‐(3‐Amino‐3‐carboxypropyl)uridine, m^1^G: 1‐Methylguanosine, acp^3^D: 3(3‐Amino‐3‐carboxypropyl)‐5,6‐dihydrouridine, m^1^I: *N*^1^‐inosine or 1‐methylinosine, D: dihydrouridine, Ψm: 2′‐O‐methylpseudouridine, m^5^C: 5‐Methylcytidine, m^2,2^G: *N*^2^,*N*^2^‐dimethylguanosine, m^1^A: *N*^1^‐methyladenosine or 1‐methyladenosine, mchm^5^U: 5‐Carboxyhydroxymethyluridine methyl ester, mcm^5^U: 5‐Methoxycarbonylmethyluridine, m^1^ψ: 1‐Methylpseudouridine , ho^5^U: 5‐Hydroxyuridine, ncm^5^Um: 5‐Carbamoylmethyl‐2′‐O‐methyluridine, I: inosine, Cm: 2′‐O‐methylcytidine, m^3^C: 3‐Methylcytidine, Gm: 2′‐O‐methylguanosine, yW-58: 7‐Aminocarboxypropylwyosine methyl ester, Q: Queuosine, Am: 2′‐O‐methyladenosine, cm^5^U: 5‐Carboxymethyluridine, mimG: Methylwyosine, OHyW: Hydroxywybutosine, m^1^Am: *N*^1^,2′‐O‐dimethyladenosine, imG: wyosine, ms^2^t^6^A: 2‐Methylthio‐*N*^6^‐threonylcarbamoyl‐adenosine)

**Figure 8.**
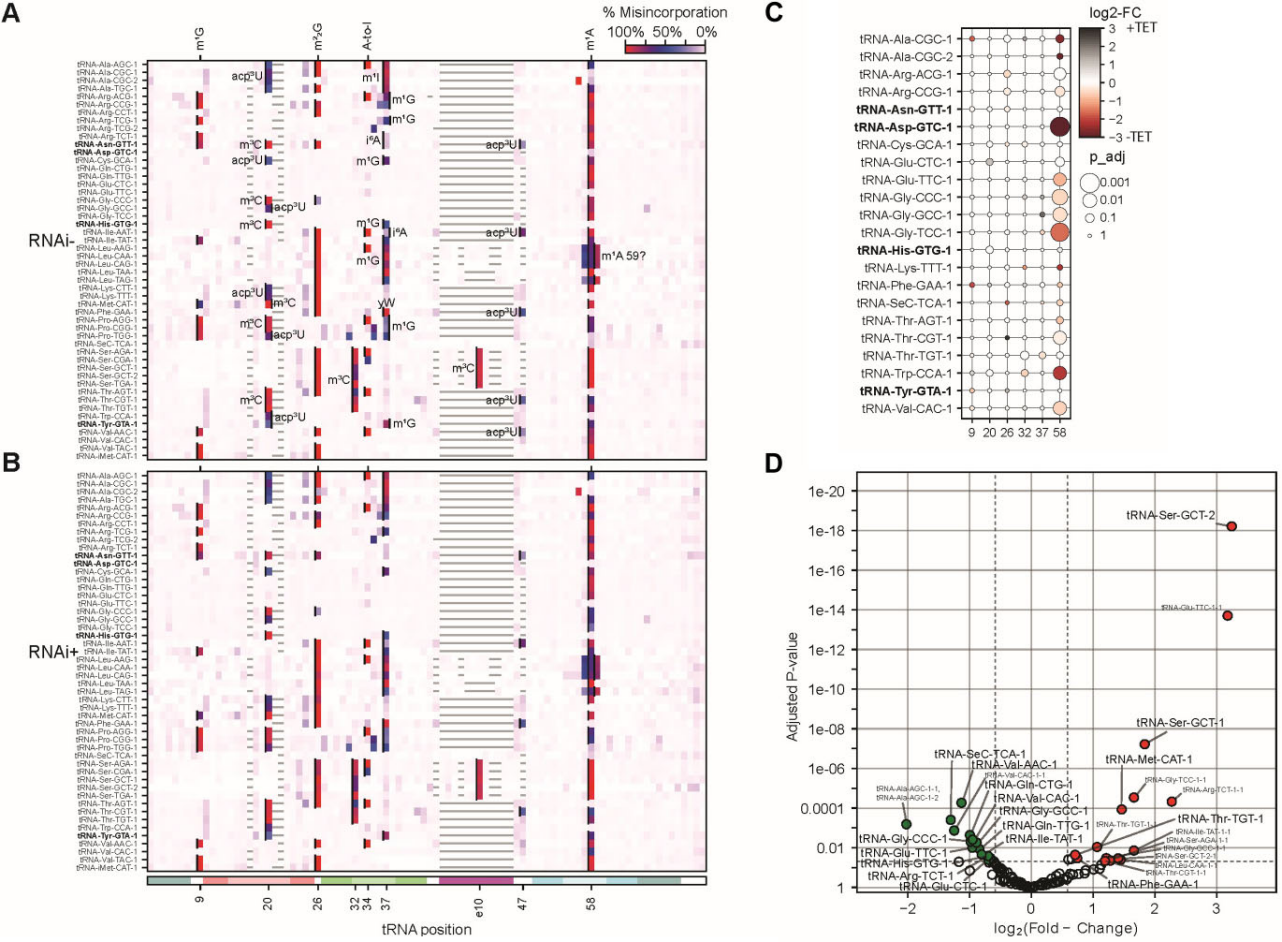
OTTR sequencing reveals m^1^A_58_ modification is decreased in a subset of tRNA upon TbLa downregulation. (**A**) Reverse Transcription (RT) % misincorporation across all *T. brucei* tRNA isodecoders in tRNA samples from TbLa RNAi uninduced (RNAi-) or (**B**) TbLa RNAi induced (RNAi+) cells. (**C**) Fold-change difference for % RT misincorporation for a subset of tRNA positions on select tRNAs (color spectrum log_2_ scale). TGT substrate tRNAs, which can be modified with Q_34_, are indicated in bold text. Circle areas indicate adjusted *P*-value thresholds as indicated. (**D**) The volcano plot comparing the log_2_ fold change of tRNA species between RNAi induced (RNAi+) and uninduced (RNAi-) conditions. The red dots indicate the tRNA species with a significant increase (*P*< 0.05) above a fold change threshold of 1.5 (log_2_(0.59)) and green dots indicate the tRNA species with a significant decrease (*P*< 0.05) below a fold change threshold of 1.5 (log_2_(0.59)) when TbLa is downregulated (RNAi+). Pre-tRNA species are labeled in smaller text while mature tRNA species are labeled in bigger text.

Given the observed effect of TbLa on Q_34_ formation, we further explored if this phenomenon is specific to Q_34_ or could also impact other tRNA modifications. To this end, total tRNA was purified from TbLa RNAi induced and uninduced cells, followed by digestion to nucleosides for global modification profiling by LC-MS/MS. The results show a significant increase in Q_34_ levels upon TbLa knockdown as previously seen on APB gels. The levels of ms^2^t^6^A, Am, Gm, cm^5^U, and OHyW modifications were also significantly increased while a decrease was observed with pseudouridine (ψ), hm^5^C, m^1^G, t^6^A, ncm^5^U, m^1^I, m^6^t^6^A, D, m^6^A, acp^3^U, and i^6^A. (Fig. 7). These results indicate that TbLa downregulation has a broad effect on the tRNA modification landscape beyond Q_34_ formation. However, one of the caveats with total nucleoside analysis using LC-MS/MS is that in cases where a particular modification occurs in different tRNAs (isoacceptors or isodecoders), changes in the observed abundance of a modification on a single specific tRNA species may be obscured by the lack of change on the rest of the tRNA population bearing the same modification. This all depends on the absolute concentration of each tRNA species amongst the sampled group. To explore the impact of TbLa on modifications of specific tRNAs and at specific sites, we implemented OTTR-seq comparing modification levels between RNA samples from TbLa RNAi-induced and uninduced cells. Alas, OTTR-seq is not perfect and it is limited to modifications which lead to a particular nucleotide mis-incorporation signature during reverse transcription. Thus, OTTR-seq permits quantification of up to 7–9 modifications at single-nucleotide resolution including m^2^G, m^2^_2_G, m^1^G, m^1^A, m^1^I, m^3^C and acp^3^U/D/ψ [63, 64]. Among the subset of modifications above, that are detectable via OTTR-seq, only m^1^A_58_ levels were significantly decreased (P value < 0.01) in some but not all tRNAs upon TbLa downregulation (Fig. 8A, B and C). The observed decrease in m^1^A_58_ for select tRNAs is reminiscent of what has been described in other organisms, and could result from the observed RNA chaperone function of TbLa [33, 34, 36, 37, 41]. It is possible that the changes in modifications observed with either technique (be it increases or decreases) were due to relative changes in the concentration of various transcript. To rule out the possibility, we performed northern blots probing for particular tRNAs and we observed not significant changes in tRNA levels upon TbLa downregulation by RNAi (Supplementary Fig. S5). As expected, one exception was elongator tRNA^Met^, which decreased in levels upon TbLa RNAi as has been previously reported [6]. Likewise, estimating tRNA abundance based on read-count during OTTR-seq showed that with the exception of few species (i.e. tRNA^Ser^_GCT-2_), in a majority of cases transcript levels were comparable between the RNAi induced and uninduced samples (Fig. 8D). But notably, for reasons that are not currently clear, elongator tRNA^Met^ does not appear to change in levels using OTTR-seq.

## Discussion

Post-transcriptional RNA modifications occur in most types of RNAs in cells, but the largest chemical diversity is found in tRNA [71–73]. tRNA modifications are catalyzed by numerous enzymes, some performing bewildering chemistry [74]. Implied in such chemical diversity is an array of metabolites that can be used as both substrates and co-factors for modification reactions, effectively connecting tRNA modification to metabolism and in turn to translational outputs [48, 72, 75]. In fact, fluctuations in the modification content of tRNAs, in response to environmental changes, have been documented for over 50 years [76–80]. These include responses to changes in temperature [78], amino acid availability [48, 77], and more recently across a range of phosphate concentrations in growth media [81]. Likely, the number of external factors that affect modification levels does not end with this brief list and examples will continue to increase as the field progresses; less clear are the molecular underpinnings that lead to modification dynamics. We, and others, have shown that intracellular transport informs modification levels, for example, the existence of competing rates between tRNA export and modification in the nucleus [48, 51].

Clearly, modifications can be affected by changes in the levels of modification enzymes and/or tRNAs themselves, but beyond this, tRNAs interact with a multitude of factors in their path from maturation to function. In eukaryotes for example, in the nucleus tRNAs interact with enzymes that mature their 5′ and 3′ ends, a subset of tRNA modification enzymes, tRNA splicing factors (in many organisms), tRNA export factors, etc. Following nuclear export, mature tRNAs interact with another subset of modification enzymes, aminoacyl tRNA synthetases, the translation machinery and eventually with factors that lead to their degradation and recycling. Any of these interactions could potentially affect modification levels. However, for all the known interactions, the complete repertoire of proteins that, for one reason or another, bind tRNA is not currently known.

Perhaps, no “professional” tRNA-binding protein (a protein that is not an enzyme) has been better studied than the La protein, which is well-known to bind tRNAs containing a 3′-poly U tail, resulting from pol III transcription [24, 25]. Bayfield and Maraia has demonstrated using *in vitro* binding assays that La also binds mature tRNA with high affinity to support their argument that La makes contact with the tRNA body in addition to 3′ trailer while chaperoning pre-tRNA [47]. However, the biological significance of such observations was not formally established. In this work we showed that downregulation of TbLa increases Q_34_ levels to almost 100% in all the TGT tRNA substrates (tRNA^Tyr^, ^-His^, ^-Asp^, and ^-Asn^). Among these, tRNA^Tyr^, the only intron-containing tRNA in *T. brucei*, gets modified with Q_34_ in the nucleus following retrograde transport after undergoing cytoplasmic splicing [49]. Prior to this work, we showed that the version of tRNA^Tyr^ that undergoes retrograde transport to the nucleus bears a mature 3′ end (devoid of a poly-U tail) [49, 82], supporting the view that at least for tRNA^Tyr^ the observed Q_34_ increase during TbLa downregulation is independent of TbLa poly-U binding function. To our knowledge, this is the first evidence for a La protein impacting an end-mature tRNA *in vivo*, and in doing so affecting the levels of a modification.

We explored several non-mutually exclusive models for how TbLa may influence Q_34_ levels in tRNA. In a previous report, we showed that increased dwell time of tRNAs in the nucleus, during conditions of reduced amino acid availability, led to increase in their Q_34_ content [48], suggesting that nuclear export and Q_34_ modification have competing rates which contribute to the observed 40–60% steady-state levels of Q_34_. Downregulation of TbLa had little impact on the dwell time of tRNAs in the nucleus, ruling out the possibility that the observed increase in Q_34_ was due to a slowdown in nuclear export caused by the absence of TbLa. We also considered the alternative hypothesis that TbLa may compete with TGT for binding fully spliced and 3′ end matured tRNA^Tyr^, as well as the other tRNAs that receive Q_34_ in the nucleus. In this model, downregulation of TbLa increases the amount of free tRNA substrate available for TGT leading to the observed increases in Q_34_. To test this hypothesis, we analyzed deletion mutants of the various RNA binding domains of La. First, we performed *in vitro* binding studies and confirmed that only LaM and RRM1 are important for mature tRNA binding. We then used the LaM and RRM1 mutants, separately, to complement the La RNAi cell line; in both cases, the levels of Q_34_ in tRNA remained high, phenocopying what was observed with the downregulation of TbLa alone. An alternative explanation for the observed phenotype in the absence of TbLa is that under normal conditions, TbLa itself interacts with TGT regulating its activity. However, we did not observe a stable interaction between TbLa and TGT in immunoprecipitation experiments (Supplementary Fig. S4). An additional question is, why in the presence of TbLa only 40–60% of those tRNAs get modified. This could be related to the relative concentrations of TbLa and TGT, in addition to the aforementioned competing rates of export. Taken together, the data supports our proposal that tRNA binding by TbLa serves as a key determinant of Q_34_ levels in tRNA. Along these lines, our findings are in agreement with the observation that *in vitro*, La can occlude access of the anticodon loop to chemical modification by kethoxal [7], with one significant difference, in our studies deletions of RRM2α or the SBM domain of TbLa had little effect on Q_34_ levels. It is possible that our mutations did not completely destroy the intrinsically disordered region of TbLa. However, the most likely explanation is that the various La domains act in concert to regulate modification levels and that indeed there are idiosyncratic differences between TbLa and La from other organisms, for example that of *S. cerevisiae*, the subject of the aforementioned study [7].

A dichotomy in the importance of genuine La in different organisms has been highlighted, whereby genuine La is essential in organisms where La has two RNA-binding motifs (RRM1 and RRM2) [8]. These include Humans and *T. brucei*, but in certain other cases, where La only has one RRM, it is not essential (i.e. *S. cerevisiae, S. pombe*) [8, 9]. Despite these observations it is not fully understood why La is essential. RRM2α has been suggested as the reason for essentiality [8]. We found that RRM2α of TbLa is dispensable for its essential function, thus ruling out the suggestion above. Despite its high degree of evolutionary conservation, it is not clear what the general function of RRM2α is. It remains possible that this domain makes contacts with tRNA and facilitates binding even though it is not a functional determinant for essentiality. This is supported by the cooperative binding behavior observed in binding curves of TbLa, and for the LaMΔ and RRMΔ TbLa mutants.

The ways that TbLa impacts modification may not be limited to our simple competition model. LC-MS/MS analysis of total nucleosides in the presence or absence of TbLa showed its role as a more global regulator of tRNA modification and not strictly a specific regulator of Q_34_ content. Some modifications significantly decreased, such as t^6^A, m^6^t^6^A, ncm^5^U and m^1^G; these are precursors or derivatives of modifications that significantly increased upon TbLa downregulation, for example, ms^2^t^6^A, cm^5^U and OHyW [73, 76, 83, 84]. This observation suggests that TbLa is important in maintaining balance in complex modification pathways and fine-tuning the levels of numerous tRNA modifications. Interestingly, all the modifications that significantly increased upon TbLa knockdown except for Am are found in the anticodon loop of tRNA. Gm and cm^5^U occur at position 34 similar to Q while OHyW and ms^2^t^6^A occur at position 37 [73, 76, 83, 84]. It is worth investigating if any of these modifications are regulated by TbLa in a similar mechanism to Q_34_. It is also possible that some of these modifications change in a Q_34_-dependent fashion similar to m^5^C_38_ modification in tRNA^Asp^ in *S. pombe*, human cells and mice [85–87]. Additionally, six out of eleven modifications that significantly decreased (t^6^A, m^6^t^6^A, i^6^A, m^6^A, m^1^I and m^1^G) are found in position 37 of anticodon loop while pseudouridine and ncm^5^U also occurs in the anticodon at position 34 [73, 76].

Changes in levels of modifications observed in the anticodon loop may have direct and complex effects on translation, not limited to selection of a specific synonymous codon over another. It is also possible that the modifications that decrease are dependent on the known chaperoning activity of La [36, 37, 39–42] and as such those effects may be more prevalent in structurally “troublesome tRNAs”, while tRNAs which easily fold naturally may not be under such dependency. OTTR-seq revealed that m^1^A_58_ went down in a subset of tRNA isoacceptors, most significantly in tRNA^Gly^_TCC_ and surprisingly in tRNA^Asp^_GTC_ (one of the Q-containing tRNAs). A_58_ methylation occurs in pre-tRNA and is important for tRNA folding and structural stability [2, 32–35], thus we speculate that in this particular case, TbLa does not serve as a competitor but may provide chaperone activity. Alternatively, TbLa may regulate the levels of the TRM6/61 enzyme itself.

The limitations of total nucleoside analysis by LC-MS/MS are that whereas global changes in modifications can be assessed, the signal for such changes becomes suppressed with modifications that occur in many tRNA species but change only in a small subset. Our analysis of tRNA modification changes via OTTR-seq, which permits single tRNA/ single-nucleotide resolution, circumvents the limitations imposed by total nucleoside analysis on quantification of the specific tRNA modifications that are detectable via OTTR-seq. Exemplifying this, the clear effect on m^1^A_58_ modification that is observed via OTTR-seq, is not apparent in the net change of m^1^A levels quantified via LC-MS/MS based total nucleoside analysis. Further, although the modifications like i^6^A, Acp^3^U, m^1^G, m^1^I are seen significantly decreased via total nucleoside analysis by LC-MS/MS, they are not seen significantly decreased below the P value threshold of 0.01 via OTTR-seq. The significant decrease of these modifications in the total nucleoside analysis could be due to the combinatorial effect of minor changes in individual tRNA species that do not appear as significant via OTTR-seq. Additionally, high sensitivity of LC-MS/MS allows detection of imbalances in modifications such as the family of modifications related to the m^1^G_37_ ‘core’ modification such as wybutosine and derivatives (i.e. yW, OHyW). Even though this position is identified as modified via OTTR-seq, this technique cannot formally differentiate between wybutosine (and derivatives) and m^1^G, both generate similar nucleotide mis-incorporation signatures during reverse transcription. The two different techniques for assessing tRNA modification levels are thus complementary, both with inherent benefits and limitations, which further emphasizes the need for technological improvements in new methods that can detect RNA modifications with high sensitivity as well as specificity [88].

The La protein can be described as an evolutionary hallmark of eukaryotes due to its conservation throughout the eukaryotic domain of life [8]. The early La protein that emerged in ancestral eukaryotes diverged into a family of La related proteins (LARPs) specialized for different substrates and functions [3, 4, 9]. Remarkably, phylogenetically deep-rooted *T. brucei* possesses only one La protein while multiple LARPs are present in other non-early diverging eukaryotes [3]. This suggests that the more ancestral TbLa may carry out multiple roles that are otherwise split amongst multiple LARPs in other systems. Interestingly, LARP7, the protein that has most similarities with genuine La proteins [9], has been linked to promoting 2′-*O*-methylation in U6 snRNA in *S. pombe* and mammals which is required for pre-mRNA splicing [89–91]. Presented with this work is one of the few instances where a member of the LARP family is involved in regulating RNA modifications. Our study also demonstrates for the first the time a role for the La protein in affecting the function of end-matured tRNAs *in vivo*. Collectively, the present work constitutes a pivotal finding for understanding how a tRNA-binding protein globally affects modification levels.

## Supplementary Material

gkaf594_Supplemental_File

## Data Availability

The data underlying this article are available in the article and in its online supplementary material.

## Notes

Present address: CEITEC-Central European Institute of Technology, Brno University of Technology, Purkyňova 656/123, Brno 61200, Czech Republic

Present address: Laboratory of Cell Biology, Center for Cancer Research, National Cancer Institute, National Institutes of Health, Bethesda, Maryland 20892, United States

## References

[1] Lerner MR, Boyle JA, Hardin JA et al. Two novel classes of small ribonucleoproteins detected by antibodies associated with lupus erythematosus. Science. 1981; 211:400–2. 10.1126/science.6164096. 6164096

[2] Hendrick JP, Wolin SL, Rinke J et al. Rosmall cytoplasmic ribonucleoproteins are a subclass of La ribonucleoproteins: Further characterization of the Ro and La small ribonucleoproteins from uninfected mammalian cells. Mol Cell Biol. 1981; 1:1138–49. 6180298 10.1128/mcb.1.12.1138-1149.1981 PMC369740

[3] Bousquet-Antonelli C, Deragon JM A comprehensive analysis of the La-motif protein superfamily. RNA. 2009; 15:750–64. 10.1261/rna.1478709. 19299548 PMC2673062

[4] Deragon J-M Distribution, organization an evolutionary history of La and LARPs in eukaryotes. RNA Biol. 2021; 18:159–67. 10.1080/15476286.2020.1739930. 32192383 PMC7928011

[5] Kerkhofs K, Garg J, Fafard-Couture É et al. Altered tRNA processing is linked to a distinct and unusual La protein in Tetrahymena thermophila. Nat Commun. 2022; 13:7332 10.1038/s41467-022-34796-3. 36443289 PMC9705548

[6] Arhin GK, Shen S, Pérez IF et al. Downregulation of the essential Trypanosoma brucei La protein affects accumulation of elongator methionyl-tRNA. Mol Biochem Parasitol. 2005; 144:104–8. 10.1016/j.molbiopara.2005.06.006. 16055205

[7] Kucera NJ, Hodsdon ME, Wolin SL An intrinsically disordered C terminus allows the la protein to assist the biogenesis of diverse noncoding RNA precursors. Proc Natl Acad Sci USA. 2011; 108:1308–13. 10.1073/pnas.1017085108. 21212361 PMC3029687

[8] Blewett NH, Maraia RJ La involvement in tRNA and other RNA processing events including differences among yeast and other eukaryotes. Biochim Biophys Acta. 2018; 1861:361–72. 10.1016/j.bbagrm.2018.01.013. 29397330

[9] Maraia RJ, Mattijssen S, Cruz-Gallardo I et al. The La and related RNA-binding proteins (LARPs): structures, functions, and evolving perspectives. WIREs RNA. 2017; 8:10.1002/wrna.1430 10.1002/wrna.1430. PMC5647580 28782243

[10] Lahr RM, Mack SM, Héroux A et al. The La-related protein 1-specific domain repurposes HEAT-like repeats to directly bind a 5’TOP sequence. Nucleic Acids Res. 2015; 43:8077–88. 10.1093/nar/gkv748. 26206669 PMC4652764

[11] Yang R, Gaidamakov SA, Xie J et al. La-related protein 4 binds poly(A), interacts with the poly(A)-binding protein MLLE domain via a variant PAM2w motif, and can promote mRNA stability. Mol Cell Biol. 2011; 31:542–56. 10.1128/MCB.01162-10. 21098120 PMC3028612

[12] Merret R, Martino L, Bousquet-Antonelli C et al. The association of a La module with the PABP-interacting motif PAM2 is a recurrent evolutionary process that led to the neofunctionalization of la-related proteins. RNA. 2013; 19:36–50. 10.1261/rna.035469.112. 23148093 PMC3527725

[13] Fonseca BD, Zakaria C, Jia JJ et al. La-related protein 1 (LARP1) represses terminal oligopyrimidine (TOP) mRNA translation downstream of mTOR complex 1 (mTORC1). J Biol Chem. 2015; 290:15996–6020. 10.1074/jbc.M114.621730. 25940091 PMC4481205

[14] Mattijssen S, Kozlov G, Gaidamakov S et al. The isolated La-module of LARP1 mediates 3’ poly(A) protection and mRNA stabilization, dependent on its intrinsic PAM2 binding to PABPC1. RNA Biol. 2021; 18:275–89. 10.1080/15476286.2020.1860376. 33292040 PMC7928023

[15] Ranjan A, Mattijssen S, Charlly N et al. The short conserved region-2 of LARP4 interacts with ribosome-associated RACK1 and promotes translation. Nucleic Acids Res. 2025; 53:gkaf053 10.1093/nar/gkaf053. 39898547 PMC11788930

[16] Dock-Bregeon AC, Lewis KA, Conte MR The La-related proteins: structures and interactions of a versatile superfamily of RNA-binding proteins. RNA Biol. 2021; 18:178–93. 10.1080/15476286.2019.1695712. 31752575 PMC7928045

[17] Intine RV, Dundr M, Misteli T et al. Aberrant nuclear trafficking of La protein leads to disordered processing of associated precursor tRNAs. Mol Cell. 2002; 9:1113–23. 10.1016/S1097-2765(02)00533-6. 12049746

[18] Bayfield MA, Kaiser TE, Intine RV et al. Conservation of a masked nuclear export activity of La proteins and its effects on tRNA maturation. Mol Cell Biol. 2007; 27:3303–12. 10.1128/MCB.00026-07. 17308035 PMC1899964

[19] Simons FHM, Broers FJM, Van Venrooij WJ et al. Characterization of cis-acting signals for nuclear import and retention of the La (SS-B) autoantigen. Exp Cell Res. 1996; 224:224–36. 10.1006/excr.1996.0132. 8612699

[20] Marchetti MA, Tschudi C, Kwon H et al. Import of proteins into the trypanosome nucleus and their distribution at karyokinesis. J Cell Sci. 2000; 906:899–906. 10.1242/jcs.113.5.899. 10671379

[21] Bayfield MA, Yang R, Maraia RJ Conserved and divergent features of the structure and function of La and La-related proteins (LARPs). Biochim Biophys Acta. 2010; 1799:365–78. 10.1016/j.bbagrm.2010.01.011. 20138158 PMC2860065

[22] Intine RV, Dundr M, Vassilev A et al. Nonphosphorylated Human La antigen interacts with nucleolin at nucleolar sites involved in rRNA biogenesis. Mol Cell Biol. 2004; 24:10894–904. 10.1128/MCB.24.24.10894-10904.2004. 15572691 PMC533991

[23] Rinke J, Steitz JA Precursor molecules of both human 5S ribosomal RNA and transfer RNAs are bound by a cellular protein reactive with anti-La Lupus antibodies. Cell. 1982; 29:149–59. 10.1016/0092-8674(82)90099-X. 7105180

[24] Stefano JE Purified lupus antigen la recognizes an oligouridylate stretch common to the 3′ termini of RNA polymerase III transcripts. Cell. 1984; 36:145–54. 10.1016/0092-8674(84)90083-7. 6607117

[25] Teplova M, Yuan YR, Phan AT et al. Structural basis for recognition and sequestration of UUUOH 3′ temini of nascent RNA polymerase III transcripts by La, a rheumatic disease autoantigen. Mol Cell. 2006; 21:75–85. 10.1016/j.molcel.2005.10.027. 16387655 PMC4689297

[26] Kessler AC, Maraia RJ The nuclear and cytoplasmic activities of RNA polymerase III, and an evolving transcriptome for surveillance. Nucleic Acids Res. 2021; 49:12017–34. 10.1093/nar/gkab1145. 34850129 PMC8643620

[27] Zhou S, Van Bortle K The Pol III transcriptome: basic features, recurrent patterns, and emerging roles in cancer. WIREs RNA. 2023; 14:e1782 10.1002/wrna.1782. 36754845 PMC10498592

[28] Maraia RJ, Kenan DJ, Keene JD Eukaryotic transcription termination factor La mediates transcript release and facilitates reinitiation by RNA polymerase III. Mol Cell Biol. 1994; 14:2147–58. 8114745 10.1128/mcb.14.3.2147-2158.1994 PMC358575

[29] Yoo CJ, Wolin SL The yeast La protein is required for the 3’ endonucleolytic cleavage that matures tRNA precursors. Cell. 1997; 89:393–402. 10.1016/S0092-8674(00)80220-2. 9150139

[30] Foldynová-Trantírková S, Paris Z, Sturm NR et al. The *T**rypanosoma brucei* La protein is a candidate poly(U) shield that impacts spliced leader RNA maturation and tRNA intron removal. Int J Parasitol. 2005; 35:359–66. 10.1016/j.ijpara.2004.12.012. 15777912

[31] Huang Y, Bayfield MA, Intine RV et al. Separate RNA-binding surfaces on the multifunctional la protein mediate distinguishable activities in tRNA maturation. Nat Struct Mol Biol. 2006; 13:611–8. 10.1038/nsmb1110. 16799560

[32] Ohira T, Miyauchi K, Sakaguchi Y et al. Precise analysis of modification status at various stage of tRNA maturation in Saccharomyces cerevisiae. Nucleic Acids Symp Ser. 2009; 53:301–2. 10.1093/nass/nrp151. 19749380

[33] Calvo O, Cuesta R, Anderson J et al. GCD14p, a repressor of GCN4 translation, cooperates with Gcd10p and Lhp1p in the maturation of initiator methionyl-tRNA in saccharomyces cerevisiae. Mol Cell Biol. 1999; 19:4167–81. 10.1128/MCB.19.6.4167. 10330157 PMC104376

[34] Kadaba S, Krueger A, Trice T et al. Nuclear surveillance and degradation of hypomodified initiator tRNA met in S. cerevisiae. Genes Dev. 2004; 18:1227–40. 10.1101/gad.1183804. 15145828 PMC420349

[35] Smoczynski J, Yared MJ, Meynier V et al. Advances in the structural and functional understanding of m1A RNA modification. Acc Chem Res. 2023; 57:429–38. 10.1021/acs.accounts.3c00568. PMC10882958 38331425

[36] Copela LA, Chakshusmathi G, Sherrer RL et al. The La protein functions redundantly with tRNA modification enzymes to ensure tRNA structural stability. RNA. 2006; 12:644–54. 10.1261/rna.2307206. 16581807 PMC1421099

[37] Vakiloroayaei A, Shah NS, Oeffinger M et al. The RNA chaperone La promotes pre-TRNA maturation via indiscriminate binding of both native and misfolded targets. Nucleic Acids Res. 2017; 45:11341–55. 10.1093/nar/gkx764. 28977649 PMC5737608

[38] Porat J, Vakiloroayaei A, Remnant BM et al. Crosstalk between the tRNA methyltransferase Trm1 and RNA chaperone La influences eukaryotic tRNA maturation. J Biol Chem. 2023; 299:105326 10.1016/j.jbc.2023.105326. 37805140 PMC10652106

[39] Pannone BK, Xue D, Wolin SL A rolefor the yeast La protein in U6 snRNP assembly: evidence that the La protein is a molecular chaperone for RNA polymerase III transcripts. EMBO J. 1998; 17:7442–53. 9857199 10.1093/emboj/17.24.7442 PMC1171088

[40] Wolin SL, Cedervall T The La protein. Annu Rev Biochem. 2002; 71:375–403. 10.1146/annurev.biochem.71.090501.150003. 12045101

[41] Chakshusmathi G, Kim SD, Rubinson DA et al. A La protein requirement for efficient pre-tRNA folding. EMBO J. 2003; 22:6562–72. 10.1093/emboj/cdg625. 14657028 PMC291820

[42] Naeeni AR, Conte MR, Bayfield MA RNA chaperone activity of Human La protein is mediated by variant RNA recognition motif. J Biol Chem. 2012; 287:5472–82. 10.1074/jbc.M111.276071. 22203678 PMC3285324

[43] Maraia RJ, Intine RV La protein and its associated small nuclear and nucleolar precursor RNAs. Gene Expr. 2002; 10:41–57. 11868987 PMC5977531

[44] Bayfield MA, Vinayak J, Kerkhofs K et al. La proteins couple use of sequence-specific and non-specific binding modes to engage RNA substrates. RNA Biol. 2019; 18:168–77. 30777481 10.1080/15476286.2019.1582955 PMC7928037

[45] Gogakos T, Brown M, Garzia A et al. Characterizing Expression and Processing of Precursor and Mature Human tRNAs by Hydro-tRNAseq and PAR-CLIP. Cell Rep. 2017; 20:1463–75. 28793268 10.1016/j.celrep.2017.07.029 PMC5564215

[46] Vinayak J, Marrella SA, Hussain RH et al. Human La binds mRNAs through contacts to the poly(A) tail. Nucleic Acids Res. 2018; 46:4228–40. 10.1093/nar/gky090. 29447394 PMC5934636

[47] Bayfield MA, Maraia RJ Precursor-product discrimination by la protein during tRNA metabolism. Nat Struct Mol Biol. 2009; 16:430–7. 10.1038/nsmb.1573. 19287396 PMC2666094

[48] Dixit S, Kessler AC, Henderson J et al. Dynamic queuosine changes in tRNA couple nutrient levels to codon choice in Trypanosoma brucei. Nucleic Acids Res. 2021; 49:12986–99. 10.1093/nar/gkab1204. 34883512 PMC8682783

[49] Kessler AC, Kulkarni SS, Paulines MJ et al. Retrograde nuclear transport from the cytoplasm is required for tRNATyr maturation in T. brucei. RNA Biology. 2018; 15:528–36. 10.1080/15476286.2017.1377878. 28901827 PMC6103694

[50] Kulkarni S, Rubio MAT, Hegedusová E et al. Preferential import of queuosine-modified tRNAs into Trypanosoma brucei mitochondrion is critical for organellar protein synthesis. Nucleic Acids Res. 2021; 49:8247–60. 10.1093/nar/gkab567. 34244755 PMC8373054

[51] Hegedűsová E, Kulkarni S, Burgman B et al. The general mRNA exporters Mex67 and Mtr2 play distinct roles in nuclear export of tRNAs in Trypanosoma brucei. Nucleic Acids Res. 2019; 47:8620–31. 10.1093/nar/gkz671. 31392978 PMC6794378

[52] Marchetti MA, Tschudi C, Kwon H et al. Import of proteins into the trypanosome nucleus and their distribution at karyokinesis. J Cell Sci. 2000; 113:899–906. 10.1242/jcs.113.5.899. 10671379

[53] LaCount DJ, Bruse S, Hill KL et al. Double-stranded RNA interference in Trypanosoma brucei using head-to-head promoters. Mol Biochem Parasitol. 2000; 111:67–76. 10.1016/S0166-6851(00)00300-5. 11087917

[54] Shi H, Djikeng A, Mark T et al. Genetic interference in Trypanosoma brucei by heritable and inducible double-stranded RNA. RNA. 2000; 6:1069–76. 10.1017/S1355838200000297. 10917601 PMC1369981

[55] ten Asbroek ALMA, Mol CAAM, Kieft R et al. Stable transformation of *Trypanosoma brucei*. Mol Biochem Parasitol. 1993; 59:133–42. 10.1016/0166-6851(93)90014-O. 8515775

[56] Chomczynski P, Sacchi∼ N Single-step method of RNA isolation by acid guanidinium thiocyanate-phenol-chloroform extraction. Anal Biochem. 1987; 162:156–9. 2440339 10.1006/abio.1987.9999

[57] Cirzi C, Tuorto F Analysis of queuosine tRNA modification using APB Northern blot assay. Methods Mol Biol. 2021; 2298:217–30. 34085248 10.1007/978-1-0716-1374-0_14

[58] Spears JL, Gaston KW, Alfonzo JD Analysis of tRNA editing in native and synthetic substrates. Methods Mol Biol. 2011; 718:209–26. 21370051 10.1007/978-1-61779-018-8_13

[59] Ross R, Cao X, Yu N et al. Sequence mapping of transfer RNA chemical modifications by liquid chromatography tandem mass spectrometry. Methods. 2016; 107:73–8. 10.1016/j.ymeth.2016.03.016. 27033178 PMC5014671

[60] Ross RL, Yu N, Zhao R et al. Automated identification of modified nucleosides during HRAM-LC-MS/MS using a metabolomics ID workflow with neutral loss detection. J Am Soc Mass Spectrom. 2023; 34:2785–92. 10.1021/jasms.3c00298. 37948765 PMC11587168

[61] Das U, Shuman S Mechanism of RNA 2′,3′-cyclic phosphate end healing by T4 polynucleotide kinase-phosphatase. Nucleic Acids Res. 2013; 41:355–65. 10.1093/nar/gks977. 23118482 PMC3592404

[62] Watkins CP, Zhang W, Wylder AC et al. A multiplex platform for small RNA sequencing elucidates multifaceted tRNA stress response and translational regulation. Nat Commun. 2022; 13:2491 10.1038/s41467-022-30261-3. 35513407 PMC9072684

[63] Upton HE, Ferguson L, Temoche-Diaz MM et al. Low-bias ncRNA libraries using ordered two-template relay: serial template jumping by a modified retroelement reverse transcriptase. Proc Natl Acad Sci USA. 2021; 118:1–10. 10.1073/pnas.2107900118. PMC8594491 34649994

[64] Holmes AD, Howard JM, Chan PP et al. tRNA Analysis of eXpression (tRAX): a tool for integrating analysis of tRNAs, tRNA-derived small RNAs, and tRNA modifications. Methods Enzymol. 2025; 711:103–23. 10.1101/2022.07.02.498565.39952700

[65] Igloi GL, Kössel H Affinity electrophoresis for monitoring terminal phosphorylation and the presence of queuosine in RNA. Application of polyacrylamide containing a covalently bound boronic acid. Nucleic Acids Res. 1985; 13:6881–98. 10.1093/nar/13.19.6881. 2414733 PMC322011

[66] Intine RV, Tenenbaum SA, Sakulich AL et al. Differential phosphorylation and subcellular localization of La RNPs associated with precursor tRNAs and translation-related mRNAs. Mol Cell. 2003; 12:1301–7. 10.1016/S1097-2765(03)00429-5. 14636586

[67] Dong G, Chakshusmathi G, Wolin SL et al. Structure of the La motif: a winged helix domain mediates RNA binding via a conserved aromatic patch. EMBO J. 2004; 23:1000–7. 10.1038/sj.emboj.7600115. 14976553 PMC380972

[68] Brown KA, Sharifi S, Hussain R et al. Distinct dynamic modes enable the engagement of dissimilar ligands in a promiscuous Atypical RNA recognition motif. Biochemistry. 2016; 55:7141–50. 10.1021/acs.biochem.6b00995. 27959512

[69] Fan H, Goodier JL, Chamberlain JR et al. 5′ Processing of tRNA precursors can Be modulated by the Human La antigen phosphoprotein. Mol Cell Biol. 1998; 18:3201–11. 10.1128/MCB.18.6.3201. 9584161 PMC108902

[70] Shan F, Mei S, Zhang J et al. A telomerase subunit homolog La protein from *Trypanosoma brucei* plays an essential role in ribosomal biogenesis. FEBS J. 2019; 286:3129–47. 10.1111/febs.14853. 30993866

[71] Björk GR, Hagervall TG Transfer RNA modification. EcoSal Plus. 2005; 1:10–1128. 10.1128/ecosalplus.4.6.2. 26443508

[72] Jackman JE, Alfonzo JD Transfer RNA modifications: nature's combinatorial chemistry playground. WIREs RNA. 2013; 4:35–48. 10.1002/wrna.1144. 23139145 PMC3680101

[73] McCown PJ, Ruszkowska A, Kunkler CN et al. Naturally occurring modified ribonucleosides. WIREs RNA. 2020; 11:e1515 10.1002/wrna.1595. PMC7694415 32301288

[74] Iwata-Reuyl D An embarrassment of riches: the enzymology of RNA modification. Curr Opin Chem Biol. 2008; 12:126–33. 10.1016/j.cbpa.2008.01.041. 18294973 PMC2430154

[75] Zaborske JM, Bauer DuMont VL, Wallace EWJ et al. A nutrient-driven tRNA modification alters translational fidelity and genome-wide protein coding across an animal genus. PLoS Biol. 2014; 12:e1002015 10.1371/journal.pbio.1002015. 25489848 PMC4260829

[76] Helm M, Alfonzo JD Posttranscriptional RNA modifications: playing metabolic games in a cell's chemical legoland. Chem Biol. 2014; 21:174–85. 10.1016/j.chembiol.2013.10.015. 24315934 PMC3944000

[77] Fournier MJ, Webb E, Kitchingman GR General and specific effects of amino acid starvation on the formation of undermodified Escherichia coli phenylalanine tRNA. BBA Sect Nucleic Acids Protein Synth. 1976; 454:97–113. 10.1016/0005-2787(76)90358-0 791374

[78] Kowalak JA, Dalluge JJ, McCloskey JA et al. The role of posttranscriptional modification in stabilization of transfer RNA from hyperthermophiles. Biochemistry. 1994; 33:7869–76. 10.1021/bi00191a014. 7516708

[79] Benko AL, Vaduva G, Martin NC et al. Competition between a sterol biosynthetic enzyme and tRNA modification in addition to changes in the protein synthesis machinery causes altered nonsense suppression. Proc Natl Acad Sci USA. 2000; 97:61–6. 10.1073/pnas.97.1.61. 10618371 PMC26616

[80] Chan CTY, Dyavaiah M, DeMott MS et al. A quantitative systems approach reveals dynamic control of tRNA modifications during cellular stress. PLoS Genet. 2010; 6:e1001247 10.1371/journal.pgen.1001247. 21187895 PMC3002981

[81] Hehenberger E, Guo J, Wilken S et al. Phosphate limitation responses in marine green algae are linked to reprogramming of the tRNA epitranscriptome and codon usage bias. Mol Biol Evol. 2023; 40:msad251 10.1093/molbev/msad251. 37987557 PMC10735309

[82] D’Almeida GS, Casius A, Henderson JC et al. tRNATyr has an unusually short half-life in *Tr**ypanosoma brucei*. RNA. 2023; 29:1243–54. 37197826 10.1261/rna.079674.123 PMC10351884

[83] Kang BI, Miyauchi K, Matuszewski M et al. Identification of 2-methylthio cyclic N6-threonylcarbamoyladenosine (ms2ct6A) as a novel RNA modification at position 37 of tRNAs. Nucleic Acids Res. 2017; 45:2124–36. 10.1093/nar/gkw1120. 27913733 PMC5389704

[84] Sample PJ, Kořený L, Paris Z et al. A common tRNA modification at an unusual location: the discovery of wyosine biosynthesis in mitochondria. Nucleic Acids Res. 2015; 43:4262–73. 10.1093/nar/gkv286. 25845597 PMC4417183

[85] Ehrenhofer-Murray AE Cross-talk between Dnmt2-dependent tRNA methylation and queuosine modification. Biomolecules. 2017; 7:14 10.3390/biom7010014. 28208632 PMC5372726

[86] Tuorto F, Legrand C, Cirzi C et al. Queuosine-modified tRNAs confer nutritional control of protein translation. EMBO J. 2018; 37:e99777 10.15252/embj.201899777. 30093495 PMC6138434

[87] Müller M, Legrand C, Tuorto F et al. Queuine links translational control in eukaryotes to a micronutrient from bacteria. Nucleic Acids Res. 2019; 47:3711–27. 10.1093/nar/gkz063. 30715423 PMC6468285

[88] Alfonzo JD, Brown JA, Byers PH et al. A call for direct sequencing of full-length RNAs to identify all modifications. Nat Genet. 2021; 53:1113–6. 10.1038/s41588-021-00903-1. 34267373

[89] Wang X, Li ZT, Yan Y et al. LARP7-Mediated U6 snRNA modification ensures splicing fidelity and spermatogenesis in mice. Mol Cell. 2020; 77:999–1013. 10.1016/j.molcel.2020.01.002. 32017896

[90] Hasler D, Meduri R, Bąk M et al. The Alazami Syndrome-associated protein LARP7 guides U6 small nuclear RNA modification and contributes to splicing robustness. Mol Cell. 2020; 77:1014–31. 10.1016/j.molcel.2020.01.001. 32017898

[91] Porat J, Slat VA, Rader SD et al. The fission yeast methyl phosphate capping enzyme Bmc1 guides 2′-O-methylation of the U6 snRNA. Nucleic Acids Res. 2023; 51:8805–19. 10.1093/nar/gkad563. 37403782 PMC10484740

