# Supplementary material for: Key RNA-binding domains in the La protein establish tRNA modification levels in Trypanosoma brucei
Source: Nucleic Acids Res. 2025 Jul 10;53(13):gkaf594. doi: 10.1093/nar/gkaf594 (PMC12242769; doi:10.1093/nar/gkaf594)
Supplement: gkaf594_Supplemental_File [file gkaf594_supplemental_file.pdf]

Key RNA-binding domains in the La protein establish tRNA modification levels in *Trypanosoma brucei*

Lankani Gunaratne[1,2,3], Henry Moore[4], Nicholas Albaum[3], Ananth Casius[2,3], Jeremy Henderson[5], Alan Kessler[6], Eva Hegedűsová[7], Sneha Kulkarni[7], Henry Arthur[2], Robert L. Ross[8], Zdeněk Paris[7,9], Richard Maraia[6], Todd M. Lowe[4] and Juan D. Alfonzo[3]*

[1] The Ohio State Biochemistry Program and The Center for RNA Biology, [2] The Ohio State University, Columbus, Ohio 43210, USA.
[3] Department of Molecular Biology, Cell Biology and Biochemistry and the Brown RNA Center, 225 Dyer Street, Brown University, Providence, Rhode Island 02903, USA.
[4] Department of Biomolecular Engineering, University of California, Santa Cruz, Santa Cruz, California 95064, USA.
[5] New England Biolabs Inc, 240 County Road Ipswich, MA 01938-2723.
[6] Section on Molecular and Cell Biology, Eunice Kennedy Shriver National Institute of Child Health and Human Development (NICHD), National Institutes of Health (NIH), Bethesda, Maryland 20892, USA.
[7] Institute of Parasitology, Biology Centre, Czech Academy of Sciences, České Budějovice, Czech Republic.
[8] Thermo Fisher Scientific, Lexington, Massachusetts 04241, USA.
[9] Faculty of Science, University of South Bohemia, České Budějovice, Czech Republic.

* To whom correspondence should be addressed. Tel: +1 614 859 0551; Email: juan_alfonzo@brown.edu

Supplementary Material

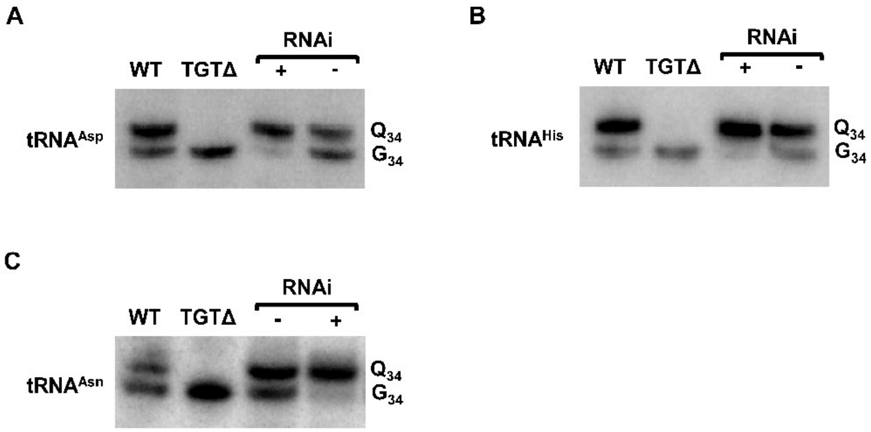

Supplementary Figure 1. Knock down of TbLa increases Q_{34} levels in tRNA. APB-Northern blots with RNA samples from WT cells, TbTGT knockout cells (TGTΔ), La RNAi- and RNAi+ cells (Day 5), probed for mature (**A**) $tRNA^{Asp}$, (**B**) $tRNA^{His}$ and (**C**) $tRNA^{Asn}$.

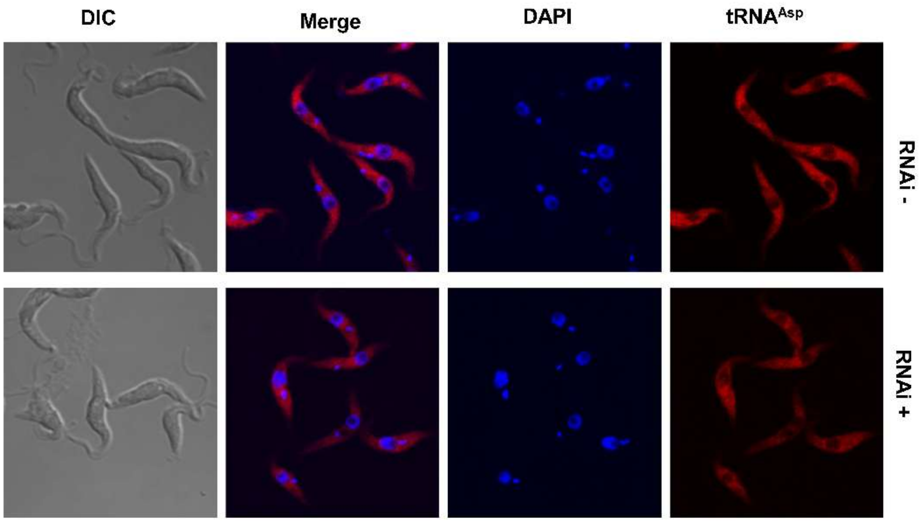

Supplementary Figure 2. The increase of Q_{34} levels in tRNA upon TbLa knockdown is not due to increased nuclear dwell time of tRNA. Florescence in situ hybridization of La RNAi- and RNAi+ cells, probed for $tRNA^{Asp}$.

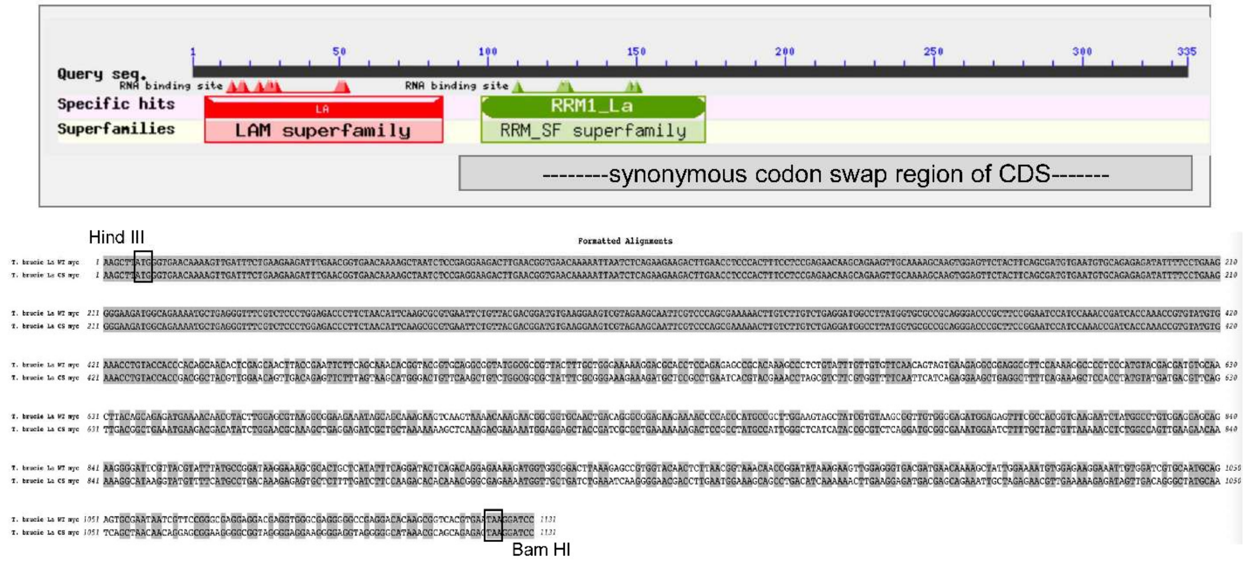

Supplementary Figure 3. Recoded TbLa gene (TbLaRec) was constructed by swapping the codons of the TbLa coding sequence with synonymous codons in the region that is targeted by La RNAi. This is shown in the alignment of WT TbLa gene (Top) with TbLaRec gene (down).

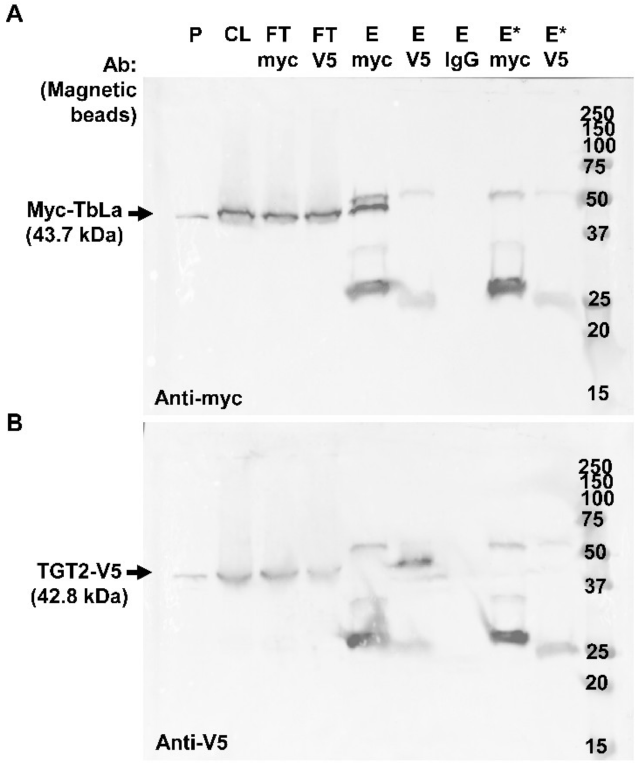

Supplementary Figure 4. The immunoprecipitation (IP) of Myc-TbLa and TbTGT2-V5 from *T. brucei* cells in which TbLa and TbTGT2 subunit of TbTGT complex are in situ tagged with a N terminal Myc tag and a C terminal V5 tag, respectively, using magnetic beads conjugated with respective antibodies. (**A**) shows the western blot with anti-myc antibody and (**B**) shows the western blot with anti-V5 antibody. In the lanes; P: pellet, CL: clear lysate, FT: flowthrough and E: elutions and '*' indicates the IPs from the WT cells as controls.

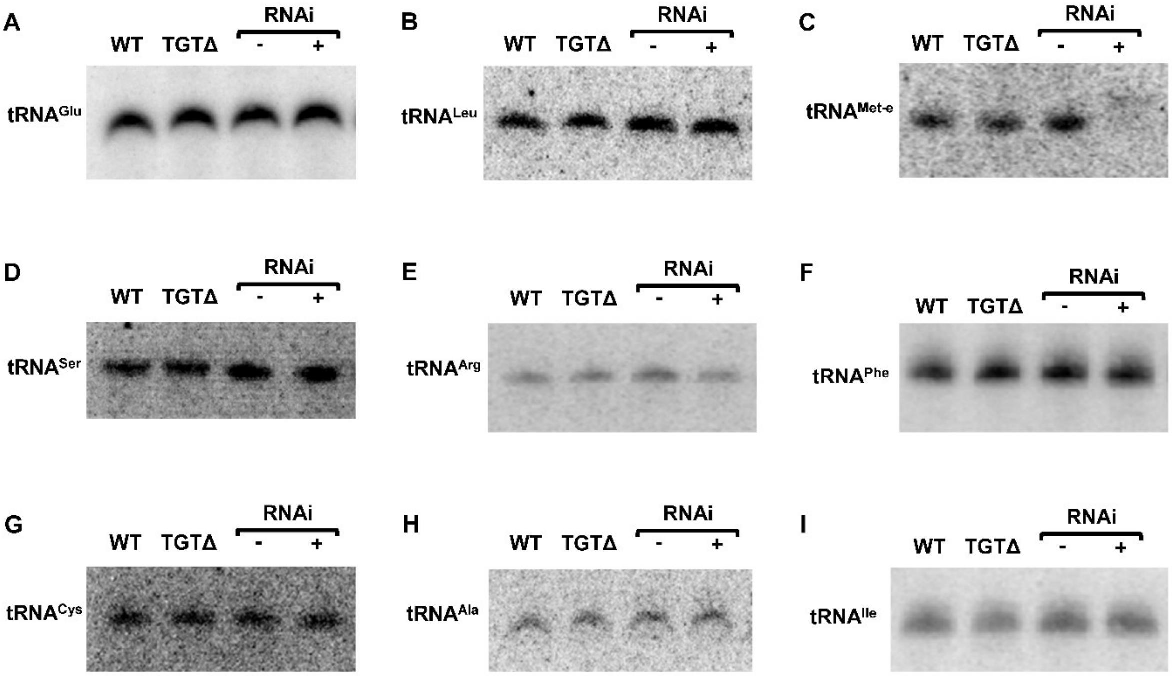

Supplementary Figure 5. Northern blots with RNA samples from WT cells, TbTGT knockout cells (TGTΔ), La RNAi- and RNAi+ cells (Day 5), probed for mature (**A**) $tRNA^{Glu}$, (**B**) $tRNA^{Leu}$, (**C**) $tRNA^{Met\text{-}e}$, (**D**) $tRNA^{Ser}$, (**E**) $tRNA^{Arg}$, (**F**) $tRNA^{Phe}$, (**G**) $tRNA^{Cys}$, (**H**) $tRNA^{Ala}$, and (**I**) $tRNA^{Ile}$
